# Food-Grade Delivery Systems for Hepatoprotective Functional Foods: From Rational Design and Delivery Mechanisms to Industrial Processing and Nutritional Intervention

**DOI:** 10.3390/foods15101713

**Published:** 2026-05-13

**Authors:** Jieyu Wang, Ying Wang, Lingjun Tong, Guoyan Liu, Jixian Zhang, Xin Xu, Chaoting Wen

**Affiliations:** 1 College of Food Science and Engineering, Yangzhou University, Yangzhou 225127, China; 13196683768@163.com (J.W.); 13861650354@163.com (Y.W.); liugy@yzu.edu.cn (G.L.); zjx@yzu.edu.cn (J.Z.); xuxin@yzu.edu.cn (X.X.); 2 Medical Science and Technology Innovation Center, Shandong First Medical University & Shandong Academy of Medical Sciences, Jinan 250117, China; ljtong@sdfmu.edu.cn

**Keywords:** food-grade delivery systems, functional foods, liver protection, bioavailability, food processing adaptability

## Abstract

The liver is a crucial metabolic organ in humans and is susceptible to oxidative stress, which can have an adverse impact on human health. Nutritional intervention with food-derived bioactive substances has the potential to improve liver health. However, their application in functional foods is limited by low oral stability and bioavailability. Therefore, a food-grade oral delivery system is required to enhance their stability and utilization efficiency. This review summarizes the research progress on the use of foodborne bioactive substances through food-grade oral delivery systems for nutritional intervention in liver oxidative stress. Firstly, this review introduces the physiological basis of liver-enriched active substances in food and the design principles of food-grade carriers. Furthermore, we summarize the types of delivery systems, including protein-based systems, polysaccharide and protein–polysaccharide composite systems, and lipid and emulsion systems, as well as emerging food-derived structural carriers. Additionally, we outline the methods for evaluating liver exposure, such as simulated digestion, intestinal transport, and hepatocyte uptake. Finally, we discuss the potential applications of machine learning in carrier design and process optimization, and analyze challenges, including large-scale production, sensory quality, and food regulations. This review provides a comprehensive theoretical and technical foundation for the development of food-grade oral delivery systems, aiming to bridge the gap between advanced delivery technologies and practical industrial applications in the functional food sector. The insights presented are expected to accelerate the development of next-generation liver health-promoting foods with high bioavailability and stable nutritional effects.

## 1. Introduction

As an important metabolic organ of the human body, the liver is the main place for nutrient metabolism and detoxification, and plays a central role in maintaining metabolic homeostasis [1]. Various dietary and environmental factors can induce oxidative stress in the liver, leading to the overproduction of reactive oxygen species (ROS). This imbalance results in metabolic dysfunction, including lipid deposition and oxidative damage [2,3]. Health problems related to liver oxidative stress have increased consumer demand for liver-protective foods. Nutritional intervention has attracted increasing attention in food science and nutrition [4].

Food-derived bioactive substances, including polyphenols (such as resveratrol and curcumin) [5], carotenoids (such as astaxanthin) [6], and bioactive peptides [7], are widely regarded as key functional components of liver health-promoting functional foods due to their excellent antioxidant and anti-inflammatory properties. Polyphenolic substances such as resveratrol and curcumin reduce liver oxidative stress by scavenging ROS and activating the Nrf2-mediated antioxidant defense pathway [8]. Carotenoids such as astaxanthin exhibit strong singlet oxygen quenching ability and have been shown to reduce lipid peroxidation and inflammation in the liver [9]. However, due to poor oral stability, low solubility, gastrointestinal degradation and significant first-pass effect, the hepatic bioavailability of these bioactive substances is low, thereby limiting their physiological efficacy. Bioactive peptides have also been explored as hepatoprotective food-derived substances, but their oral delivery faces similar stability challenges. Recent reviews have summarized traditional and stimuli-responsive peptide delivery systems [10]. Rational carrier design strategies can enhance their health effects [11]. However, compared with polyphenolic substances, there are still few studies on food-grade peptide delivery systems, and quantitative verification remains limited.

As an advanced food processing and formulation technology, food-grade oral delivery systems have emerged as a key solution to addressing these problems. Distinct from drug delivery systems, food-grade delivery systems are constructed from food ingredients that comply with Generally Recognized as Safe (GRAS) standards. The core design goal of these systems is to enhance the gastrointestinal stability and bioavailability of food-grade bioactive substances while ensuring compatibility with traditional food processing technologies and the sensory quality of food products [12]. By designing the structure of the delivery carrier, these systems can prevent the degradation of bioactive substances in food in the gastrointestinal tract, achieve controlled release at target absorption sites, and improve hepatic bioavailability, thereby enhancing their nutritional effects on liver health. The integration of nanotechnology and food-grade delivery systems has further promoted the development of these systems. Nanocarriers have unique size effects and surface properties, which can effectively enhance the efficiency of intestinal epithelial transport and liver passive enrichment [13]. Simultaneously, the integration of food-grade delivery systems with precision nutrition enables the customization of personalized nutritional intervention strategies for individuals at varying risks of hepatic oxidative stress. Common food-grade oral delivery carriers are all based on raw materials from the food industry, including protein-based carriers (plant proteins, animal proteins) [14], polysaccharides and protein–polysaccharide composite systems (pectin, alginate) [15], and liposome and emulsion-based systems (food-grade liposomes, nanoemulsions) [16,17], as well as emerging food-derived structural materials [18,19]. These carriers not only exhibit excellent biocompatibility and safety but can also regulate gastrointestinal digestion through structural design, thereby effectively improving the hepatic exposure of bioactive substances and their intervention effects on liver oxidative stress.

**Figure 1:**
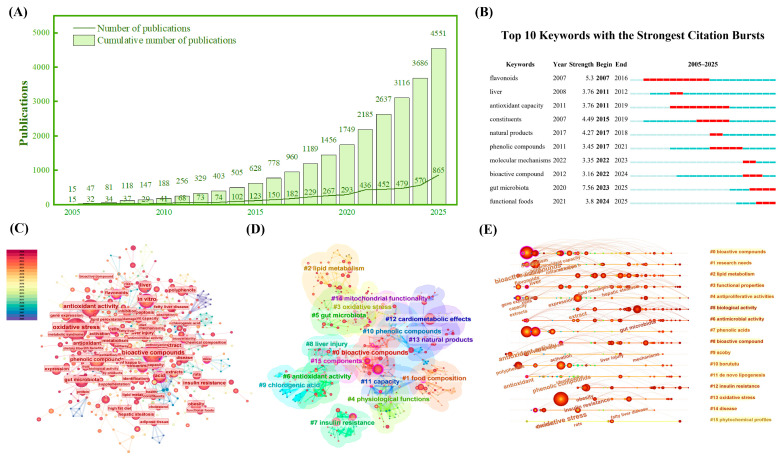
(**A**) Annual and cumulative number of publications on food-grade oral delivery systems for nutritional intervention in liver oxidative stress in the field of Food Science & Technology (2005–2025). Data were retrieved from the Web of Science Core Collection using the following topic keywords: “food-grade oral delivery system”, “bioactive substances”, “liver oxidative stress”, and “nutritional intervention”, and were restricted to the research area of Food Science & Technology. The knowledge structure and emerging trends in this field (2005–2025) were further analyzed using CiteSpace 6.4. The results include (**B**) top 10 keywords with the strongest citation bursts, (**C**) keyword co-occurrence network, (**D**) cluster view, and (**E**) timeline visualization. The analysis was based on 994 publications retrieved within the period from 2005 to 2025.

As shown in Figure 1, both annual and cumulative publications on food-grade oral delivery systems for liver oxidative stress have increased steadily from 2005 to 2025. In particular, a marked rise in recent years reflects growing interest in their application for nutritional intervention. This trend suggests that the field is emerging as an important focus within functional food and nutrition research.

Compared with the reviews published in recent years that mainly focus on specific categories of bioactive substances or individual delivery systems, this review provides a more comprehensive and application-oriented perspective. Previous studies have generally emphasized material selection, encapsulation efficiency, or individual biological activity assessment, while limited attention has been paid to the complete pathway from gastrointestinal digestion to liver exposure under realistic nutritional intervention scenarios. In contrast, this review introduces the concept of liver exposure efficiency as a core framework, systematically linking carrier design, gastrointestinal fate, intestinal transport, and liver uptake into a unified evaluation system. In addition, it goes beyond the traditional summary and incorporates emerging topics, including different delivery systems and data-driven design strategies, such as machine learning-assisted optimization. Importantly, this work also emphasizes the translation dimension by considering food processing compatibility, industrial scalability, and physiologically relevant dose design. These aspects provide a more practical basis for the rational development of liver-protective functional foods.

## 2. Methods

This review follows the PRISMA 2020 statement where applicable [20]. Given its mechanism-oriented nature (focusing on carrier design, gastrointestinal fate, and liver exposure rather than intervention effect sizes), a scoping review framework was adopted.

A systematic literature search was performed in the Web of Science Core Collection for the period from 2005 to 2025, using the following keywords: “food-grade oral delivery system”, “bioactive substances”, “liver oxidative stress”, and “nutritional intervention”. The search was restricted to the research area of Food Science & Technology. The last search was performed on 25 March 2026.

Eligibility criteria: Studies were included if they (1) reported food-grade delivery systems for bioactive substances, (2) addressed gastrointestinal stability, intestinal transport, or liver-related outcomes, and (3) provided original experimental data. Exclusion criteria included non-English articles, conference abstracts, and studies without relevance to liver oxidative stress or food-grade carriers.

**Figure 2:**
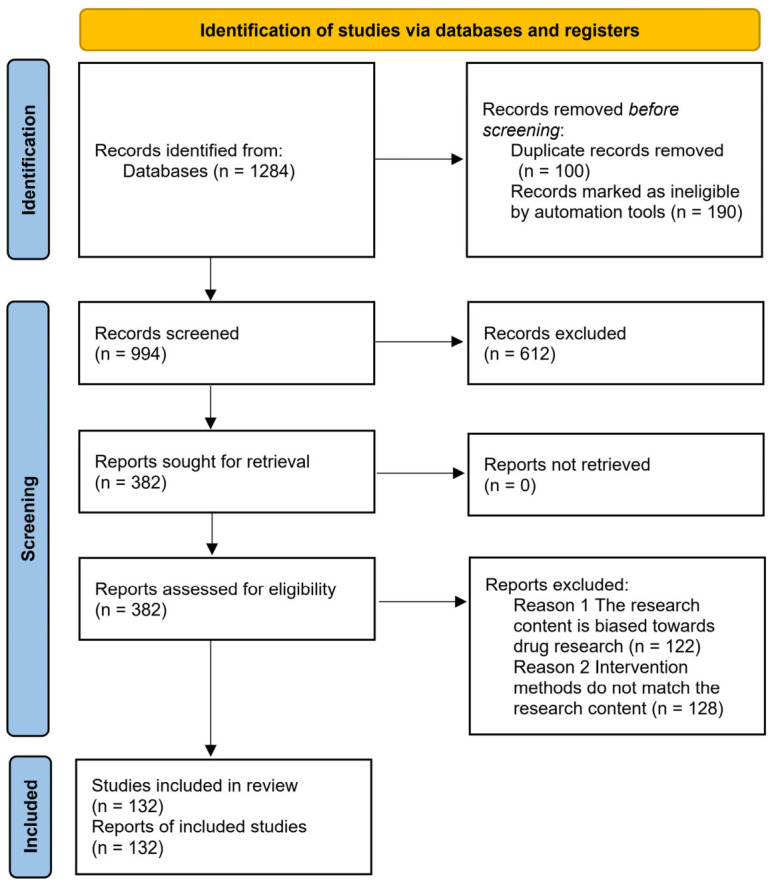
Summary of the PRISMA-based literature screening process, including identification, screening, eligibility assessment, and final inclusion stages.

Screening process: Two reviewers independently screened titles/abstracts and then full texts. Disagreements were resolved by consensus. The PRISMA flow diagram (Figure 2) summarizes the selection process. A total of 132 studies were included in the qualitative synthesis (Table S1).

Statement of compliance: The literature retrieval and screening process adhered to the PRISMA guidelines. Because this review does not involve quantitative meta-analysis or effect size estimation (e.g., risk of bias for individual studies in the traditional sense), items related to effect measures, statistical synthesis, and risk of bias assessment are not applicable.

## 3. Physiological Basis and Design Strategy of Food-Grade Oral Delivery System for Liver Health

### 3.1. The Stability of Food-Derived Bioactive Substances in the Gastrointestinal Tract and Their Bioavailability in the Liver

**Figure 3:**
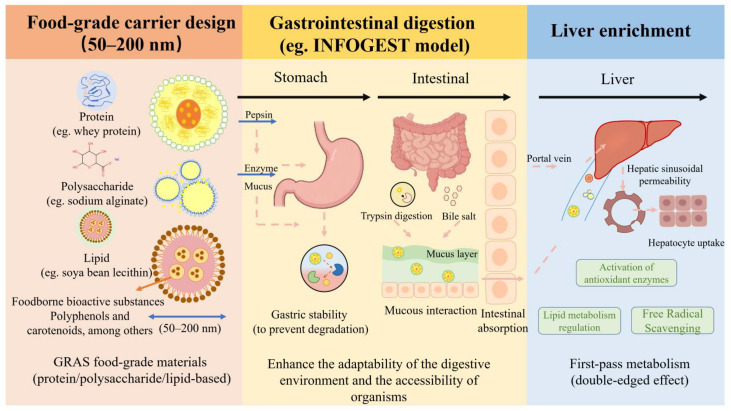
Stable gastrointestinal release and hepatocyte uptake of food-grade carriers.

Oral administration is the most commonly used administration method for functional foods. It has the advantages of convenience and personalization. However, due to the various barriers of the gastrointestinal tract, food-derived bioactive substances are difficult to be effectively delivered to the liver. As shown in Figure 3, food-grade carriers maintain structural integrity in the gastric acid and digestive enzyme environment by surface modification or the characteristics of the material itself, avoiding premature leakage or degradation of active substances. After entering the intestine, the carrier responds to pH, enzymes or specific trigger factors to achieve controlled release of active substances, thereby improving the enrichment efficiency of bioactive substances in the liver. This process enables controlled regulation of stability, release, and absorption during gastrointestinal transit, thereby improving the liver exposure of bioactive substances through physiological transport pathways.

#### 3.1.1. Multi-Barrier Constraints Affecting the Stability and Bioavailability of Food-Derived Bioactive Substances

Food-grade bioactive substances need to overcome barriers in the gastrointestinal tract after oral intake, including physical, chemical, and biological barriers. They limit the stability and absorption efficiency of food-borne bioactive components, resulting in a decrease in their utilization.

First, the physical barrier mainly comes from the gastrointestinal structure itself. The intestinal mucus layer is composed of mucins that form a dense network structure, which can capture nanocarriers or active molecules through electrostatic and hydrophobic interactions, thereby significantly reducing their ability to diffuse to the epithelial surface [21]. In addition, tight junctions between intestinal epithelial cells limit the paracellular transport of macromolecules and particles, making it difficult for most food-grade bioactive substances to effectively cross the intestinal barrier and enter the circulatory system.

Secondly, the chemical barrier mainly includes the complex physical and chemical environment in the gastrointestinal tract. Factors such as strong acidic environment in the stomach (pH 1–3), bile salts in the small intestine, and changes in ionic strength may cause structural instability, oxidative degradation, or conformational changes in bioactive substances such as polyphenols and carotenoids [14,22]. At the same time, these physical and chemical factors may also affect the integrity of the carrier structure and further accelerate the release and inactivation of active ingredients [23,24]. However, it should be noted that most of the current studies on the stability of active substances are mainly based on in vitro simulated digestion models. These models are still different from the real dietary environment in terms of pH conditions, enzyme concentration, and reaction time, which may overestimate the degree of degradation of bioactive substances to a certain extent. In addition, the interaction of proteins, polysaccharides and lipids in food systems may provide some protection to active substances.

Third, the biological barrier mainly involves the combined effects of digestive enzymes, intestinal microorganisms and metabolic processes. Digestive enzymes (such as proteases, lipases, and others) can directly degrade carrier structures or active molecules and reduce their biological activity. Gut microbiota can change the structure and function of bioactive substances through metabolic transformation [25,26]. In addition, bioactive substances enter the liver through the portal vein after intestinal absorption and undergo significant first-pass metabolism. Although this process contributes to liver-targeted exposure, excessive metabolism may also reduce their effective concentration into systemic circulation [27,28].

It is worth noting that the limiting effect of different types of barriers on food-borne active substances is not equivalent, and the degree of influence depends largely on the physical and chemical properties of active substances and the structural characteristics of carriers. For example, small molecule lipophilic substances are more susceptible to chemical degradation. Nano-scale carriers are more susceptible to mucus layer interception and epithelial barrier limitations. Therefore, the above barriers do not exist in isolation, but show the characteristics of material dependence and synergy.

#### 3.1.2. Quantitative Description of Gastrointestinal Transport and Hepatic Exposure

At present, the research on food-grade delivery systems mainly focuses on the qualitative description of gastrointestinal stability and transport routes, while quantitative characterization is still limited. In particular, the lack of kinetic data and transport parameters hinders the establishment of a predictive framework for delivery performance. To address this gap, coupled kinetic and transport models can be introduced to quantitatively describe gastrointestinal fate and liver exposure.

During gastrointestinal digestion, the release behavior of encapsulated bioactive substances can be approximated by a first-order kinetic model or a diffusion-controlled kinetic model [29]:

$$\frac{M_{t}}{M_{\infty }}=k\times t^{n}$$

where *k* denotes the release rate constant and *n* denotes the release mechanism.

The transport of bioactive substances across the intestinal epithelium is usually evaluated using the apparent permeability coefficient (*P*_app_) [30]:

$$P_{app}=\frac{dQ/dt}{A\times C_{0}}$$

where *dQ*/*dt* is the transport rate, *A* is the surface area of the epithelial monolayer, and *C*_0_ is the initial concentration.

In addition, the diffusion through the mucus layer can be described by the effective diffusion coefficient (*D*_eff_), which is strongly affected by particle size, surface charge and mucus–particle interaction. It can be expressed by liver exposure efficiency (*E*_liver_):

$$E_{liver}=\frac{{AUC}_{liver}}{{AUC}_{plasma}}$$

This reflects the relative accumulation of bioactive substances in the liver compared with the systemic circulation.

The integration of these parameters provides a multi-scale quantitative framework linking gastrointestinal release, epithelial transport and liver accumulation. The framework enables the transition from empirical design to mechanism-driven optimization of a food-grade delivery system for nutrition intervention.

Therefore, an efficient food-grade oral delivery system has become a crucial technology, which helps to improve the stability, bioavailability and liver exposure of food-grade bioactive substances in the gastrointestinal tract. Rational design must be based on the physiological characteristics of gastrointestinal digestion and liver absorption. Therefore, it is necessary to design a more intelligent food-grade carrier so that bioactive substances can be released and absorbed at the appropriate site and time. This method aims to maximize the nutritional value of bioactive substances through mild physiological pathways, which is consistent with the principle of nutritional intervention, rather than the scope of clinical treatment. Future research should further build a comprehensive evaluation system combining real food system, dynamic digestion process and in vivo verification, so as to narrow the gap between mechanism research and actual nutritional intervention effect.

### 3.2. Design Principles of Food-Grade Oral Delivery Carriers

In the development of functional foods, the design of food-grade oral carriers is subject to a variety of constraints, including gastrointestinal stability, intestinal absorption, liver exposure, food safety, processing adaptability and sensory compatibility [31]. Unlike drug nanocarriers that prioritize precise targeting and treatment outcomes, food-grade delivery systems must operate during physiological transport and meet food-related requirements, making their optimization inherently multidimensional.

**Figure 4:**
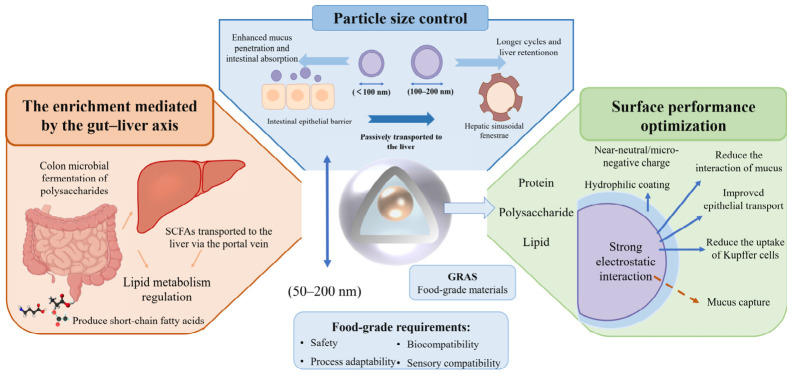
Summary of design principles of food-grade oral delivery carriers.

The performance of food-grade delivery systems is affected by key structural parameters, such as particle size, surface properties, and fermentability [32]. These parameters also affect a variety of functional indicators, including bioavailability, epithelial permeability (*P*_app_), mucus diffusion (*D*_eff_), and liver exposure efficiency (*E*_liver_). However, these parameters are often interdependent and may exhibit competing effects. Therefore, the rational design of food-grade carriers requires balancing competing performance objectives, rather than optimizing a single parameter. From this performance-oriented perspective, the following sections analyze key design strategies, including particle size regulation, surface property optimization, and gut–liver axis-mediated regulation (Figure 4).

#### 3.2.1. Particle Size Regulation: The Core Mechanism of Passive Liver Enrichment

The regulation of particle size is a key factor to achieve passive enrichment of food-grade carriers in the liver. Its design should take into account the fenestrae structure of hepatic sinusoidal endothelial cells and the transport characteristics of the gastrointestinal tract. Previous studies have shown that particles in the 50–200 nm range are optimal under specific physiological conditions [33]. However, this range should not be regarded as a general rule, as liver uptake is a complex process influenced by many factors, including protein corona formation, opsonization and hemodynamic conditions. For food-grade delivery carriers, the optimal particle size needs to be adjusted according to the composition and surface characteristics of the carrier. Smaller nanoparticles (less than 100 nm) have stronger mucin permeability and intestinal absorption efficiency, which are suitable for those active substances with poor stability in the gastrointestinal tract. Larger particles (100–200 nm) have a longer circulation time in the body and stronger retention capacity in the liver, which is conducive to continuous nutritional intervention in liver oxidative stress. [34]. When using food-grade materials (such as proteins, polysaccharides and lipids) to construct nanocarriers, the most critical issue is the uniformity and stability of particle size [35]. At the same time, the design of particle size also needs to consider the edibility of functional foods. For example, nanocarriers for transparent functional beverages need to have smaller and more uniform particle sizes to avoid turbidity and precipitation.

#### 3.2.2. Optimization of Surface Properties: Improving the Transport Efficiency of the Gastrointestinal Tract

The surface properties of food-grade carriers play a crucial role in mucus penetration and intestinal epithelial absorption [36]. By using natural food-grade components to adjust the hydrophobicity, charge characteristics or hydrophobic/hydrophilic ratio of the carrier surface, the diffusion ability of the carrier in the mucus layer can be enhanced and the efficiency of transepithelial absorption can be improved. It is generally believed that a structure with a nearly neutral or slightly negative charge on the surface is beneficial to reduce the electrostatic interaction with the negatively charged mucin fibers, thereby improving the permeability of mucus [37]. By adding natural hydrophilic food components to the surface of the carrier to change its surface structure, a hydrophilic protective layer can be formed, thereby reducing the phagocytosis of Kupffer cells and prolonging the circulation time of the carrier in the body [38]. In addition, the surface modification of food-grade carriers must follow the principle of edibility and natural origin. All modifiers must be food raw materials or food additives that meet GRAS standards, such as natural monosaccharides such as galactose and mannose, and edible plant polysaccharides. At the same time, chemical modifiers, such as targeting ligands, should be avoided to ensure the food safety of the delivery system. Surface properties also involve competition effects. Near-neutral or slightly negative surfaces reduce electrostatic interactions with mucus and improve diffusion, while positively charged particles may enhance cellular uptake but are more likely to be trapped in the mucus layer. Therefore, optimizing surface properties requires balancing mucus penetration and epithelial absorption efficiency.

#### 3.2.3. Indirect Liver Enrichment Mediated by the Gut–Liver Axis: Enhancing the Effect of Nutritional Intervention

The gut–liver axis provides an additional pathway by which the liver can be regulated using a food-grade delivery system. This is an indirect but functionally important design strategy. Unlike particle size regulation and surface optimization, which mainly affect the transport and absorption processes, this approach focuses on the use of colonic fermentation and microbiota-mediated metabolism to enhance liver-related nutritional effects.

Food-grade carriers, especially fermentable polysaccharides such as pectin and inulin, can be selectively degraded by the gut microbiota in the colon, leading to the production of short-chain fatty acids (SCFAs). These metabolites are then transported to the liver via the portal vein, where they are involved in the regulation of lipid metabolism, inflammatory response and oxidative stress. For example, Li et al. demonstrated that dietary pectin improved liver lipid metabolism and reduced oxidative stress by regulating gut microbiota composition and increasing SCFA production [39]. Tian et al. found that rice protein peptides alleviated hepatic steatosis, oxidative stress and inflammation in mice with alcoholic liver disease by regulating intestinal microflora composition and up-regulating short-chain fatty acid levels, and inhibiting fatty acid biosynthesis gene expression through the PPARγ signaling pathway [40]. In addition, inulin-type fructans have been widely reported to enhance butyrate production, inhibiting liver lipid accumulation and oxidative stress, highlighting their role in gut microbiota-mediated liver protection [41]. These findings indicate that a delivery system based on fermentable polysaccharides can not only be used as a carrier, but also as a prebiotic substrate to synergistically enhance liver-targeted nutritional intervention through the gut–liver axis [42]. This mechanism highlights the dual functional design concept of food-grade carriers. On the one hand, they are vehicles for the delivery of bioactive substances. On the other hand, they can be used as functional dietary components that interact with gut microbiota. For example, pectin-based delivery systems have been shown to enhance SCFA production and improve intestinal barrier function, thereby indirectly alleviating liver oxidative stress. Similarly, inulin carrier can regulate the composition of intestinal microflora, promote the production of beneficial metabolites, and help to improve liver metabolic homeostasis [43]. Therefore, gut–liver axis-mediated regulation should be considered as a complementary design strategy in food-grade delivery systems. This method does not directly increase the accumulation of bioactive substances in the liver, but instead enhances the protective effect of the liver through physiological metabolic pathways to achieve nutritional intervention.

The design of the carrier should be compatible with traditional food processing technologies to ensure the stability and controlled release performance of the active substance during processing, storage and consumption. At the same time, it should also be ensured that its taste and flavor will not be significantly affected. The application potential of this food-grade nano-delivery system in functional beverages and dietary supplements has shown promising prospects. In summary, comprehensive strategies, including particle size regulation, surface property optimization, edible ligand modification, and indirect enrichment through the gut–liver axis, can ensure the safety and processability of food-grade oral delivery carriers. At the same time, the gastrointestinal stability, intestinal absorption rate and liver exposure of the active substances are improved. This provides a solid technical foundation for the development of liver health-promoting functional foods with high bioavailability, stable nutritional effects and good market acceptance.

## 4. Types of Food-Grade Oral Delivery Carriers Used in Liver Health

**Figure 5:**
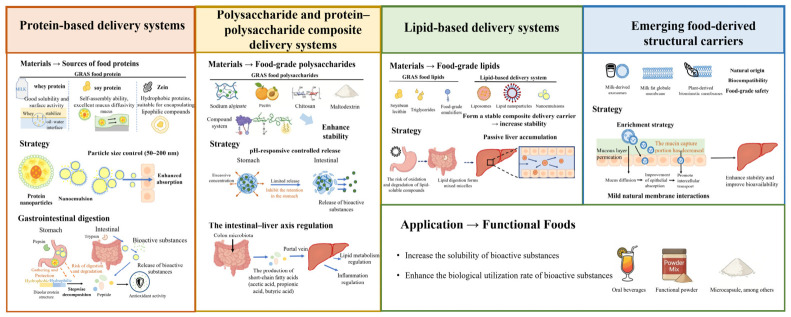
The liver enrichment strategy and functional food suitability of different food-grade oral delivery carriers.

Although a variety of food-grade delivery systems have been developed, they show significant differences in gastrointestinal stability, transport efficiency, liver exposure, and food processing adaptability. These carriers are not simply interchangeable, but have their own unique advantages and limitations due to their different structural characteristics and interactions with biological systems and food matrices. The existing food-grade oral delivery systems can be divided into protein-based systems, polysaccharides and protein–polysaccharide composite systems, lipid and emulsion systems, and emerging food-derived structural carriers (Figure 5). Different types of carriers have their own advantages in structural characteristics, processing adaptability and functional performance. Table 1 systematically summarizes the differences in encapsulation ability, structural characteristics and application scenarios of different food-derived carriers, which provides a basis for the detailed analysis of various subsequent delivery systems.

### 4.1. Protein-Based Delivery Systems

Protein-based delivery carriers have become an important direction for food-grade oral delivery research due to their biocompatibility, controllable digestibility and processability. Compared with synthetic polymers, natural proteins are more easily accepted by food regulations and can be directly used in functional foods or dietary supplement systems. Common food protein sources in the industry include whey protein, soy protein, and zein. The structure and interface properties of different proteins determine their applicable carrier types. From the perspective of structure–performance relationship, whey protein has good solubility and interfacial activity, which is suitable for constructing emulsion or nanodispersion systems, but it is prone to denaturation and aggregation in the gastric acid environment. Soybean protein has strong self-assembly ability and structural plasticity, which is suitable for constructing nanoparticles or composite systems, but its interfacial activity is relatively weak. Zein has strong hydrophobicity, which is beneficial to encapsulate fat-soluble active substances, but its water dispersibility is poor, and it usually needs to be used in combination with other hydrophilic components. Therefore, different protein types show significant performance differences between “stability–loading capacity–release behavior”, which determines their applicability in different functional food systems.

Whey protein has good solubility and interfacial activity, and can form a stable protein film at the oil–water interface [75]. Takbirgou et al. prepared nanoparticles by heating whey protein, which effectively protected casein from hydrolysis during gastrointestinal digestion and significantly improved the stability of curcumin [76]. Soybean protein has strong plasticity and self-assembly ability, which can be easily used to construct nanoparticles or composite systems [77]. Yuan et al. prepared soy protein nanoparticles with rapid mucus diffusion ability, which significantly improved the bioavailability of curcumin by enhancing cell uptake, transmembrane penetration and transcytosis [78]. These food proteins are classified as GRAS and are gradually degraded into amino acids and small peptides during digestion, leaving no non-degradable residues. Importantly, many of these hydrolyzed peptides possess inherent antioxidant, anti-inflammatory, or immunomodulatory activities, which can synergize with the encapsulated bioactive ingredients to enhance the overall nutritional efficacy of the functional food product.

The structure of proteins changes in the gastrointestinal environment, which provides a natural protection and release regulation mechanism for active substances. Under acidic conditions in the stomach, protein molecules usually undergo partial denaturation and form aggregation structures, thereby limiting the diffusion rate of active substances. After entering the small intestine, the protein network is gradually degraded under the action of trypsin, so that embedded active substances are released at the absorption site. For example, Sadiq et al. developed casein micelle microcapsules that encapsulate anthraquinones via strong hydrophobic interactions, achieving controlled release in the gastrointestinal tract [79]. Zhang et al. successfully constructed pepsin-resistant soybean peptide nanoparticles, which efficiently encapsulate curcumin through a core–shell structure to achieve intestinal controlled release, reduce lipid peroxidation and increase antioxidant enzyme levels [80].

Most food proteins are amphiphilic molecules, and their molecular structure contains hydrophilic and hydrophobic regions, which can stabilize the oil–water interface and form emulsion or nanoemulsion systems. For example, Shen et al. prepared astaxanthin nanodispersions stabilized by whey protein isolate using an emulsification–evaporation technique. The resulting nanodispersions exhibited small particle size, high encapsulation efficiency, amorphous morphology, and good digestive stability, which significantly improved astaxanthin permeability in a Caco-2 cell model [81]. Huan et al. prepared whey protein-encapsulated astaxanthin nanoemulsions with an appropriate particle size (approximately 110 nm), which significantly improved the bioavailability of astaxanthin. This was achieved by inhibiting catabolism, promoting anabolism, and regulating factors related to mitophagy and glucose metabolism [82].

The protein-based delivery system has good processing adaptability and can be prepared into different structural forms according to food type. Nanoemulsions and nanodispersions are suitable for liquid functional foods, such as functional or milk-based beverages. For example, Wang et al. demonstrated that encapsulating curcumin in α-lactalbumin nanocarriers effectively improved its stability and bioavailability in functional milk beverages [83]. Protein microcapsules prepared by spray drying are more suitable for solid products, such as meal replacement powder or dietary supplements. Wang et al. constructed a double-layer microcapsule encapsulation technology to enable probiotics to resist the harsh environment of the gastrointestinal tract and promote their adhesion and colonization in the intestine, thereby achieving effective delivery of vitamin B2 [84]. These differences in performance are essentially derived from the difference in protein molecular structure (such as hydrophobic/hydrophilic ratio, secondary structure stability, and interface behavior), which affects conformational changes, carrier stability, and active substance release behavior in the gastrointestinal environment.

At present, the research mainly focuses on the delivery of polyphenols (such as curcumin, resveratrol and quercetin), while the research on carotenoids and fat-soluble vitamins mostly stays in the stage of in vitro digestion or cell experiments. As shown in Table 1, the preparation process of protein-based carriers is relatively mature and the encapsulation efficiency is high. Its advantages lie in good biocompatibility and controllable release behavior. However, it is sensitive to pepsin and prone to precipitation in an acidic environment, resulting in poor gastric stability. Most of the current studies on protein-based delivery systems are still in the in vitro digestion model or cell experiment stage (such as Caco-2 or HepG2 model), and the results mainly reflect the potential mechanism rather than the nutritional effects under actual dietary conditions. Therefore, the direct extrapolation of these results to functional food applications should still be approached with caution, and more validation based on real food systems and in vivo studies is needed in the future. Future research may pay more attention to the high-value utilization of food processing by-product proteins, such as soybean meal protein or wheat gluten protein, to reduce production costs and improve the sustainability of the system.

### 4.2. Polysaccharide and Protein–Polysaccharide Composite Delivery Systems

Natural polysaccharides are widely used in the construction of food-grade delivery systems due to their wide availability, diverse structure and good biocompatibility. Common raw materials include sodium alginate, pectin, chitosan and maltodextrin. These polysaccharides not only exhibit gelling and emulsifying properties but can also regulate intestinal microecology and provide nutritional benefits.

Polysaccharides are highly responsive to gastrointestinal pH changes, providing a basis for designing intestinal release systems. Sodium alginate can form a dense gel structure in the acidic environment of the stomach, thereby limiting the release of active substances, while the gel gradually expands and releases the encapsulated bioactive substances under high pH conditions in the small intestine. Huang et al. prepared freeze-dried microcapsules loaded with astaxanthin using a layer-by-layer assembly technique using chitosan, sodium alginate and maltodextrin as wall materials. The encapsulation efficiency of the freeze-dried microcapsules was more than 90%, and the bioavailability of astaxanthin was significantly increased to 69 ± 1% by controlling the digestion process [85]. Rim et al. developed pH-responsive microcapsules loaded with flaxseed extract using a sodium alginate–fish gelatin matrix. The encapsulation efficiency exceeded 90%, and rapid intestinal release was achieved under simulated gastrointestinal conditions, improving bioavailability [86].

Polysaccharide-based delivery systems have received increasing attention in regulating the gut–liver axis, especially in the production of SCFAs during colonic fermentation. Fermentable polysaccharides can be utilized by intestinal microorganisms to produce SCFAs such as acetic acid, propionic acid and butyric acid. These metabolites can enter the liver through the portal vein and participate in the regulation of lipid metabolism, inflammatory response and oxidative stress. For example, Liu et al. have shown that chitosan/pectin nanoparticles loaded with astragalus polysaccharides are associated with delayed hepatic steatosis and reduced inflammation, and their effects may be related to the regulation of gut microbiota and the promotion of acetic acid production [87]. However, the relationship between SCFAs and liver health is highly complex and context dependent, and is affected by multiple factors such as gut microbiota composition, host metabolic status, and dietary background. In addition, the current evidence is mainly derived from in vitro fermentation models or animal experiments, reflecting more potential mechanisms than direct causal relationships under real dietary conditions. Therefore, SCFA-mediated effects should be considered as an auxiliary regulatory pathway rather than a deterministic mechanism. In the future, it is still necessary to further verify their functional significance in real food systems and human studies to clarify its practical application value in food-grade delivery systems.

In practical applications, in a single polysaccharide system, it is often difficult to balance structural stability and gastrointestinal-responsive release. Sodium alginate has good pH responsiveness, but its mechanical stability is weak. Chitosan has strong adhesion, but it has limited water solubility. Therefore, the construction of delivery systems through a protein–polysaccharide composite strategy has gradually become a research hotspot. Proteins and polysaccharides can form a stable composite structure through electrostatic interaction, hydrogen bonding and hydrophobic interactions. Proteins provide interfacial activity and structural support, while polysaccharides endow the system with pH responsiveness, anti-enzymatic degradation ability and intestinal microecological regulation function, thereby improving the stability of bioactive substances in the gastrointestinal tract. Dong et al. prepared rutin microcapsules via complex coacervation of soy protein isolate and chitosan hydrochloride. The microcapsules exhibited high encapsulation efficiency (90.34%) and intestinal-targeted release, significantly enhancing the antioxidant activity of the digestive products [88].

Polysaccharides and protein–polysaccharide composite systems have diverse structural forms and can be adapted to different types of functional foods. Hydrogels or microspheres based on sodium alginate and pectin are often used to construct gel-type foods [89]. They can also be prepared into powder by freeze drying or spray drying for meal replacement powder or dietary supplements [90]. Chitosan-based nanoparticles have good water dispersibility and can be applied to functional beverage or oral liquid systems [91].

Current research mainly focuses on the delivery of polyphenols and carotenoids, such as curcumin, quercetin and astaxanthin. Sodium alginate, pectin and chitosan are mostly used as raw materials. Preparation technologies have also evolved from traditional emulsification or spray drying to nanostructure construction and multi-component composite systems, such as layer-by-layer assembly or protein–polysaccharide covalent cross-linking. As shown in Table 1, the gastric protective ability of polysaccharide-based carriers is significantly improved by their resistance to gastric acid and enzymes, but the release of some polysaccharides in the intestine was incomplete or the response was too slow, which may reduce the delivery efficiency of active substances. Composite carriers are designed to integrate the advantages of each component, and achieve an ideal curve of low release in the stomach and high release in the intestine in simulated gastrointestinal fluid. However, the preparation process is complicated, the repeatability between batches is poor, and the large-scale production cost is significantly increased. Future research may focus more on the high-value utilization of food processing by-product polysaccharides (such as pectin, cereal dietary fiber), and the design of composite delivery systems that can regulate the metabolism of the gut–liver axis.

### 4.3. Lipid-Based Delivery Systems

Lipid-based delivery systems have garnered significant attention in food-grade oral delivery research due to their structural similarity to the cell membrane and their high encapsulation capacity for lipophilic bioactive substances. They are typically fabricated from food-grade lipids, such as phospholipids, triglycerides, and emulsifiers. In food-grade lipid delivery systems, common structures include liposomes, nanoemulsions, and lipid nanoparticles (LNPs). These systems are usually prepared by food processing technologies such as high-pressure homogenization or microfluidization, and exhibit good industrial feasibility.

Liposomes are composed of phospholipid bilayers, which are structurally similar to the cell membrane and exhibit good biocompatibility. Liposomes can encapsulate hydrophilic components in the core of the aqueous phase and dissolve fat-soluble substances in the phospholipid bilayer, so that synergistic delivery of multiple active substances can be achieved. For instance, Wu et al. developed phosphatidyl–agar oligosaccharide-modified liposomes for astaxanthin encapsulation, which significantly improved stability and in vitro digestibility, demonstrating the potential of food-grade liposomes to enhance the bioavailability of lipophilic nutrients in functional foods [92].

Nanoemulsions are transparent or semi-transparent systems composed of oil, water, and emulsifier, and the particle size is usually less than 200 nm. Due to their large interfacial area and good solubility, nanoemulsions can significantly improve the solubility and digestion and absorption efficiency of fat-soluble active substances. Staszewski et al. prepared fish oil emulsion by using the nano-complex formed by β-lactoglobulin and green tea polyphenols as a emulsifier, which significantly improved the stability of the emulsion and the oxidative stability of fish oil [93]. Harwansh et al. prepared nanoemulsions loaded with betulinic acid by the spontaneous nanoemulsification method. The improved solubility and oral bioavailability of betulinic acid delivered via nanoemulsion enhanced liver-protective effects [94].

LNPs are usually constructed from edible lipids and emulsifiers, with high encapsulation efficiency and good digestive stability. Unlike LNPs used for nucleic acid delivery in the pharmaceutical field, LNPs in food systems are mainly used to deliver natural active substances [95]. Li et al. prepared resveratrol LNPs, which effectively reduced hepatic lipid accumulation and oxidative stress [5]. It is very important to distinguish food-grade LNPs from drug LNP systems. Drug LNPs are usually designed for nucleic acid delivery and typically rely on synthetic or ionizable lipids to achieve high cellular uptake and endosomal escape. In contrast, food-grade LNPs are composed of edible lipids such as phospholipids, triglycerides and natural emulsifiers, which are mainly used to improve the solubility, stability and bioavailability of bioactive substances. Therefore, the design emphasizes safety, digestibility and compatibility with food processing conditions, rather than cellular uptake or endosomal escape efficiency.

Lipid and emulsion systems have a wide range of applications in food. Nanoemulsions exhibit good transparency and dispersibility. Liposomes and LNPs can be prepared into powder by spray drying. In addition, lipid carriers can also be integrated into food systems such as dairy products, chocolate or nut butter to improve the stability of fat-soluble active substances.

Current research in this area primarily focuses on the delivery of lipophilic bioactives, such as curcumin, resveratrol, and astaxanthin. Phospholipids, triglycerides, and food-grade emulsifiers are commonly used to fabricate liposomal systems. Preparation technologies have gradually evolved from thin-film dispersion or homogenization methods to the regulation of nanoscale structures. As shown in Table 1, the lipid and emulsion systems have outstanding advantages in solubilizing hydrophobic polyphenols, and the encapsulation efficiency is high. However, poor physical stability and bile salt-induced burst release limit their practical application. Lipid-based delivery systems face key limitations such as oxidative degradation and physical instability in food applications. Lipid oxidation not only reduces the stability of the encapsulated active substances, but also may produce odor, affecting the functional effect and sensory quality of the product. At the same time, environmental factors such as temperature, light and oxygen will significantly shorten shelf life. In order to improve the stability, they are often modified by adding natural antioxidants, optimizing the interface structure and spray drying. However, in industrial applications, such systems are highly sensitive to processing conditions, such as homogenization pressure, temperature, and formulation composition, which can affect particle size distribution, interface stability, and encapsulation efficiency. In addition, in real food systems, lipid carriers may also interact with proteins, polysaccharides and other components, further affecting their stability. Therefore, industrial feasibility depends not only on laboratory performance, but also on the comprehensive optimization of process scalability, system stability and storage conditions. Future research may pay more attention to the structural regulation of lipid raw materials, the application of natural emulsifiers, and the design of lipid delivery systems combined with proteins or polysaccharides, so as to further enhance the system’s stability and expand its application potential in functional foods.

### 4.4. Emerging Food-Derived Structural Carriers

Emerging food-borne structural carriers have gradually attracted attention in recent years and are considered as potential oral delivery systems. These carriers are usually derived from natural food raw materials and have good biocompatibility and safety. However, their functional performance and application feasibility in food systems are still in the exploratory stage.

Exosomes, as natural nanovesicles derived from milk or plant cells, have been used for the delivery of bioactive substances. Kumar et al. isolated exosome from the mixed homogenate of turmeric and pepper by a green one-pot method, and successfully achieved the synergistic co-encapsulation of curcumin and piperine, which significantly enhanced the anti-inflammatory activity and stability [73]. Although these studies have shown potential stability and biological activity advantages, the current evidence is mainly derived from in vitro or animal models, and their behavior in real food systems and humans remains to be clarified.

Biomimetic membrane coating technology is also used to improve the stability of the carrier and its interaction with the biological interface. Studies have shown that membrane-camouflaged nanocarriers exhibit enhanced interactions with cell membranes in vivo, promoting endocytosis and facilitating transepithelial transport and targeted release of bioactive substances [96]. However, these results are mostly based on idealized experimental conditions, and their applicability in complex gastrointestinal environments and food systems still needs to be further verified.

Emerging carriers have certain potential in mucus penetration and cell interaction, but their practical application in functional foods is still subject to factors such as large-scale preparation, batch stability, regulatory acceptance, and long-term food safety. Compared with the established systems based on proteins, polysaccharides and lipids, the emerging carriers are still in the early development stage and need to be further verified before practical application. Therefore, such carriers should be regarded as exploratory strategies, rather than mature technology paths. Future research needs to focus on functional verification under real dietary conditions and application feasibility in food processing systems.

In general, different carriers have their own advantages in structural characteristics, embedding ability and applicable food types. The protein-based system has good nutrition and processing adaptability. The polysaccharide system has advantages in intestinal response and microecological regulation. The liposome system is more suitable for the delivery of fat-soluble active substances, and the new structural carrier provides a new idea for improving bioavailability. In functional food development, carrier selection must consider the physicochemical properties of the bioactive substances, the target food matrix, and processing conditions. The carrier structure should be rationally designed to enhance the stability and bioavailability of the active ingredients while ensuring food safety and processing compatibility.

## 5. Verification Strategies for Hepatic Exposure Efficiency of Food-Grade Carriers

**Figure 6:**
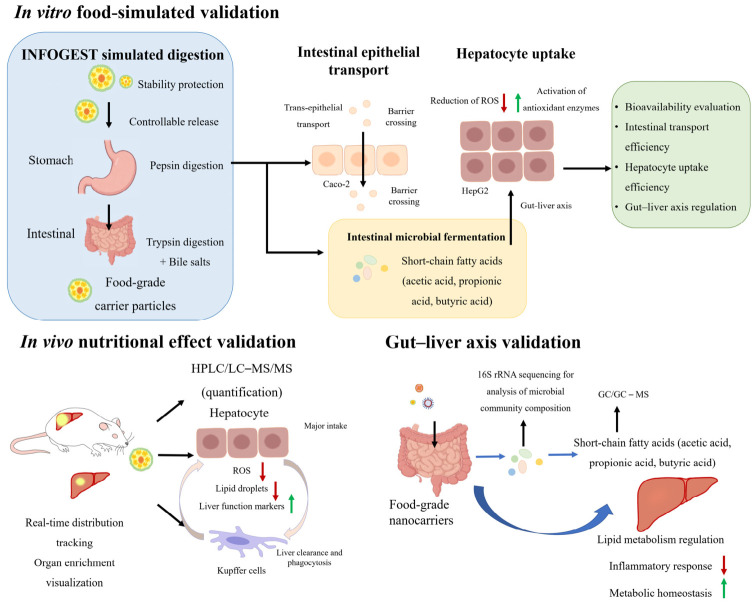
Multi-dimensional verification system for liver enrichment efficiency of food-grade carriers. Red ↑ means decline, and green ↓ means rise.

Hepatic exposure efficiency refers to the evaluation of whether food-grade oral delivery carriers can effectively deliver food-grade bioactive substances to the liver and exert liver health-promoting effects. The key is to measure the bioavailability concentration of these substances in the liver (Figure 6). Unlike precise receptor recognition in drug delivery systems, the accumulation of food-grade carriers in the liver depends mainly on the passive enrichment of their physical and chemical properties (such as particle size). This mode of action is more in line with the basic concept that functional foods promote health through nutrition. Therefore, it is very important to establish a validation strategy that can simulate the whole process from intake to hepatic exposure. It is necessary to simulate the whole process from ingestion to hepatocyte uptake in a multi-level and multi-dimensional manner, taking into account the complexity of the food system.

### 5.1. In Vitro Evaluation Strategy: Simulated Digestion, Intestinal Absorption and Hepatocyte Uptake

In vitro evaluation is an important means to study the behavior changes of food-grade bioactive substance delivery systems during digestion and absorption. This kind of method is simple and low cost, and has good repeatability by simulating the digestion process of the human gastrointestinal tract. Therefore, it is widely used in the research on functional foods and food delivery systems.

Based on the INFOGEST model, by simulating the pH environment, enzyme composition and digestion time of different stages of oral, gastric, and intestinal phases, the release kinetics and chemical morphological changes of active substances during digestion can be systematically investigated. In the study of food delivery systems, the model can also be personalized. For example, Ji et al. prepared exosomes derived from Lentinula edodes, which can be used as a natural carrier to encapsulate curcumin efficiently. By enhancing its stability, prolonging intestinal retention time and promoting the biotransformation driven by intestinal beneficial bacteria, the metabolism and biological activity of curcumin can be significantly improved [97]. These optimizations enable the in vitro digestion model to better simulate real physiological conditions. On this basis, researchers usually evaluate the protective effect of delivery carriers on bioactive components by measuring the retention rate, release curve and final bioavailability of active substances at different digestion stages.

Many studies still remain in the digestion stage itself, focusing only on the structural changes of the carrier in the gastrointestinal environment and the release behavior of active substances. The cell model can be used to evaluate the biological effects of active substances. Xiao et al. prepared extracellular polysaccharide selenium nanoparticles of Cordyceps sinensis, which effectively alleviated H_2_O_2_-induced oxidative stress in HepG2 cells by regulating antioxidant enzyme activity and inhibiting ROS production [98]. However, such segmented approaches are insufficient to fully capture the complete process of food-grade bioactive substances, spanning from intake, digestion and absorption, to intestinal epithelial transport, hepatocyte uptake, and subcellular distribution. Specifically, intestinal epithelial cell models (e.g., Caco-2) are primarily used to evaluate the transport efficiency of carriers across the intestinal barrier, whereas hepatocyte models (e.g., HepG2) mainly reflect the intracellular effects of bioactive substances after hepatic uptake. Neither of them can reproduce the continuous and dynamic process from intestinal absorption to liver uptake. Therefore, another study coupled the Caco-2 monolayer intestinal epithelial model with the HepG2 liver cell model, which can simulate the intestinal–liver transport axis in the human body [99]. The bioactive substances released by food-grade carriers after digestion are taken up by HepG2 cells after passing through the Caco-2 cell monolayer, which can simultaneously evaluate the intestinal transport efficiency and hepatocyte uptake efficiency of food-grade carriers, and more truly reflect the bioavailability of bioactive substances.

In addition to the limitations of model simplification, the physiological correlation of the doses used in in vitro studies should also be carefully considered. Many cell-based experiments use relatively high concentrations of bioactive substances to ensure observable biological effects, which may exceed what can be achieved with a normal diet of functional foods [100]. Therefore, the biological activity may be higher than the actual efficacy. From a nutritional point of view, the effective concentration of bioactive substances reaching the target organ is affected by many factors, including food matrix effect, gastrointestinal digestion, intestinal absorption and first-pass metabolism. Therefore, under ideal circumstances, the concentration of in vitro application should be associated with the estimated dietary intake level and bioavailability data to improve the transformation correlation of the research results. Future studies should combine physiologically relevant dose designs with advanced in vitro models, such as digestion–absorption coupling systems or gut–liver axis models, to better simulate in vivo exposure.

In addition to the digestion and absorption process, the gut–liver axis has also attracted much attention in the function of food-grade active substances. At present, most studies still focus on the mechanism of active substances in the single link of the intestine or liver, and lack systematic evaluation of the comprehensive role of gut microbiota metabolites in regulating liver oxidative stress, inflammatory response and lipid metabolism [101]. Studies have shown that gut microbiota can significantly affect liver oxidative stress and inflammatory response by producing SCFAs, polyphenol metabolites and other small molecule metabolites, thus playing an important role in regulating liver metabolic homeostasis [102]. For example, Liu et al. constructed Astragalus polysaccharide nanoparticles to regulate the gut–liver axis, restore the balance of gut microbiota and promote acetic acid production, effectively alleviating metabolic-related fatty liver disease [87].

In summary, by integrating an in vitro simulated digestion system, intestinal epithelial absorption model and gut–liver axis-related evaluation system, the mechanism of action of food-grade delivery carriers in digestive stability, intestinal absorption and liver function regulation can be more comprehensively revealed. The establishment of a multi-level in vitro evaluation model not only helps to improve the systematic understanding of the bioavailability process of foodborne active substances, but also provides an important theoretical basis for the development of functional foods and nutritional intervention research.

### 5.2. In Vivo Evaluation Strategy: From Distribution Tracking to Functional Verification

At present, most studies on food-derived delivery systems are still based on in vitro models, focusing on evaluating their digestive stability and cell uptake efficiency. However, the nutritional utilization process of active substances in complex body environments and their actual effects on liver function still need to be verified by in vivo studies. Through animal models, the effects of active substances on liver oxidative stress, lipid metabolism and inflammatory response can be observed, so as to more comprehensively evaluate the nutritional intervention potential of delivery carriers. Studies have shown that fluorescence labeling tracking technology is a common method to evaluate the distribution of food-grade carriers in vivo. The key requirement is to use fluorescent probes for food safety to label food-grade carriers or bioactive substances. For example, using near-infrared fluorescent dye-labeled protein nanoparticles, it was found that they could produce strong fluorescence signals in the liver region after oral administration, thus providing visual evidence of the ability of the carrier to enhance the hepatic accumulation of the encapsulated nutrients [103]. Through in vivo imaging, the migration path and organ enrichment of food-grade carriers in vivo after oral administration can be observed in real time, and the spatial and temporal distribution characteristics of carriers in the liver can be intuitively reflected.

Simple fluorescence imaging can only provide relative distribution information, and it is necessary to quantitatively detect the actual content of bioactive substances in liver tissue to accurately evaluate the liver exposure efficiency of food-grade carriers. Commonly used detection methods include high-performance liquid chromatography (HPLC) and liquid chromatography–tandem mass spectrometry (LC–MS/MS). They have the advantages of high sensitivity and good accuracy, and can detect the concentration changes of bioactive substances in liver tissue at different time points after oral administration of food-grade carriers. For example, LC–MS technology has been used to quantitatively detect the accumulation of polyphenols in liver tissue in nano-delivery systems. The results showed that compared with the free-form, nanocarriers could significantly increase the concentration of active substances in liver tissue, thereby enhancing their antioxidant or anti-inflammatory effects [104]. This quantitative evidence supports the potential of food-grade delivery systems to achieve the desired nutritional effects at lower intake doses, aligning with the principles of efficient and sustainable functional food design.

Therefore, by integrating fluorescence tracer imaging and tissue quantitative analysis, the migration law and hepatic accumulation mechanism of food-grade delivery system in vivo can be more systematically revealed. It not only helps to clarify the enrichment and action sites of active substances in the liver, but also provides a more reliable experimental basis for the structural design and functional optimization of the delivery system. In the future, the establishment of a comprehensive evaluation system combining in vivo imaging and tissue quantification will help to better understand the delivery behavior of food-grade delivery carriers in the liver and promote its application in functional foods and liver-related nutritional interventions.

### 5.3. Integration of Gut–Liver Axis Model in Verification System

With the increasing attention to the role of the gut–liver axis in metabolic regulation and liver health, the mechanisms by which food-derived delivery systems participate in liver nutrition regulation by regulating intestinal flora has also begun to receive attention. At present, most of the verification strategies still focus on the direct liver uptake of bioactive substances, and there is insufficient research on the indirect regulation mechanism of the gut–liver axis.

The in vitro intestinal flora simulated fermentation system is widely used to evaluate the metabolic behavior of food ingredients in the colon environment. Studies have shown that delivery systems based on food-grade polysaccharides or polyphenols can promote the growth of some beneficial bacteria and change the composition of microbial metabolites during fermentation. For instance, polysaccharide-based nano-delivery system can significantly promote the growth of beneficial bacteria during in vitro fermentation and change the composition of microbial metabolites, thereby regulating the metabolism of the gut–liver axis [105]. For example, Liu et al. prepared alginate microspheres containing quercetin, which can not only directionally convert polyphenols into highly active phenolic acids, but also significantly promote the formation of SCFAs and optimize the structure of gut microbiota by jointly regulating the metabolism of gut microbiota [106]. Among these metabolites, SCFAs are considered to be important signaling molecules connecting gut microbiota and host metabolism. SCFAs mainly include acetic acid, propionic acid and butyric acid, which can enter the liver through the portal vein and participate in lipid metabolism and inflammatory response regulation. Some fermentable polysaccharide or polyphenol delivery systems can increase the production level of SCFAs. These metabolites can participate in the regulation of liver lipid metabolism through related receptor signals and energy metabolism pathways [107]. For example, Bai et al. prepared sodium alginate–chitosan quercetin oral colon-targeted microspheres, which promoted SCFA production by enriching short-chain fatty acid-producing bacteria, and achieved metabolic regulation pathways mediated by the gut–liver axis to affect liver health [108]. Therefore, the change of SCFAs not only reflects the change of metabolic activity of gut microbiota, but also realizes that food-derived bioactive substances affect liver health through the gut–liver axis.

In general, the verification of liver bioavailability in food-derived delivery systems has gradually developed from a single evaluation method to a multi-level comprehensive evaluation system. By integrating an in vitro simulated digestion model, intestinal epithelial absorption model, hepatocyte uptake evaluation and gut microbiota fermentation system, the action process of food-grade carriers can be systematically analyzed from multiple levels, such as digestive stability, intestinal transport efficiency, hepatocyte uptake ability and flora metabolism regulation. At the same time, in vivo fluorescence tracing, tissue quantitative detection and liver physiological index analysis can further verify the migration path and nutritional intervention effect of the delivery system in the body. This multi-level evaluation strategy from in vitro mechanism research to in vivo functional verification provides an important research framework for systematically understanding the whole process of food-derived bioactive substances from intake to absorption to liver function.

In future studies, there is still room for further improvement in the verification system of liver bioavailability. On the one hand, it is necessary to construct a comprehensive model closer to the real physiological environment, such as a multi-organ collaborative model integrating intestinal epithelial cells, hepatocytes and the gut microbiota metabolic system, so as to more accurately simulate the dynamic regulation process of the gut–liver axis. On the other hand, with the development of multi-omics technology, metabolomics, microbiome and transcriptomics are expected to be used to systematically analyze the regulation mechanism of food-derived delivery system on gut microbiota metabolism and liver metabolic pathways. In addition, by combining in vivo imaging technology with high-sensitivity tissue quantitative analysis, the exposure level and site of action of bioactive substances in the liver can be more accurately evaluated. This provides a more solid theoretical basis for functional food development and liver health-related nutrition intervention.

## 6. The Frontier Strategy of Carrier Design: Data-Driven and Artificial Intelligence

**Figure 7:**
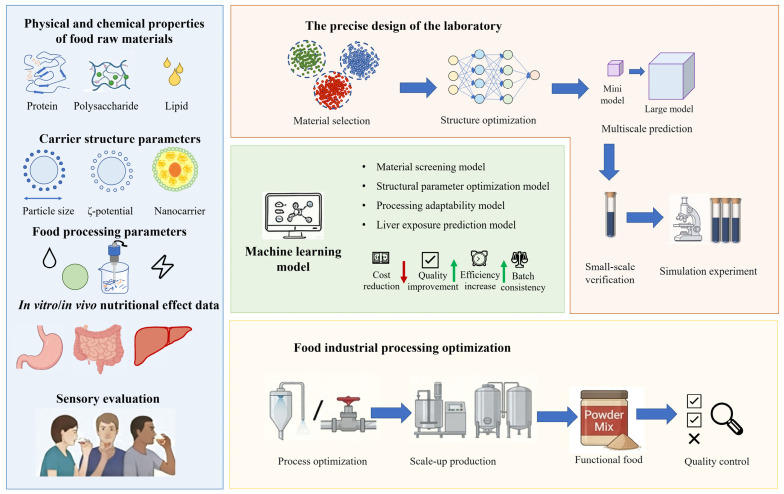
Application of data-driven and intelligent optimization in food-grade carrier design and food industry process. Red ↑ means decline, and green ↓ means rise.

With the development of data science and artificial intelligence technology, the research and development of food-grade oral delivery systems is gradually shifting from traditional empirical trials to data-driven precision design. The gastrointestinal stability, encapsulation efficiency and liver exposure level of food-grade carriers are usually affected by multiple factors such as particle size, ζ potential, carrier composition and processing conditions. There is a complex nonlinear relationship between these parameters and the nutritional intervention effect of functional foods. Machine learning (ML) can integrate multi-source data such as the structure of active substances, the physical and chemical properties of carriers, and digestion behavior to construct a structure–function prediction model, thus providing new research ideas for the structural design and process optimization of food-grade delivery systems (Figure 7).

### 6.1. The Application of Machine Learning in Carrier Design

By integrating the physicochemical properties of materials, structural parameters of carriers, and in vitro digestion and bioavailability data, the ML model is expected to construct a multi-scale prediction framework that spans molecular, structural, and processing levels, thereby promoting the rational design of food-grade delivery systems from experience-driven to data-driven.

The selection of carrier materials is the basis for the construction of food-grade delivery systems. It is necessary to comprehensively consider various factors such as food safety, physical and chemical properties, processing adaptability and sensory compatibility. Traditional screening methods usually rely on step-by-step experimental verification, which is inefficient and difficult to systematically evaluate the comprehensive performance of different materials. ML can establish a material performance prediction model by integrating the physical and chemical properties, encapsulation efficiency and digestive stability of food-grade materials such as proteins, polysaccharides and lipids, so as to achieve rapid screening and combination optimization of candidate materials. Studies have shown that the deep integration of nanotechnology and artificial intelligence is comprehensively innovating the detection methods, quality control systems and nutrition assessment paradigms of food chemistry by achieving accurate detection, targeted delivery and bioavailability improvement of food components, as well as pattern recognition and intelligent analysis based on multi-source data [109].

ML can establish a structure–function prediction model by integrating structural parameters and delivery performance data, and realize multi-objective optimization by combining genetic algorithm or particle swarm optimization [110]. For example, Rahdar et al. accurately identified the key parameters to reduce the toxicity of curcumin nanocarriers by integrating experimental data with the ML framework of physical theory, and achieved an optimal design with a loading rate of 70% [111]. In addition, the ML model can further integrate food processing parameters to predict the optimal processing conditions and reduce the experimental trial and error process, thus promoting the development of food-grade delivery systems towards industrial production.

Multi-scale structure–function prediction provides a new research perspective for the design and optimization of food-derived delivery systems. By integrating the molecular level, nanonetwork structure and macroscopic carrier construction data, a multi-scale model can be constructed to accurately simulate the dynamic behavior of active substances in the process of gastrointestinal digestion, transepithelial absorption and liver-targeted enrichment [112]. For example, molecular dynamics simulation can predict the interaction stability of polyphenols with protein or polysaccharide carriers, nanostructure characterization can evaluate the structural retention ability of carriers in gastrointestinal fluid, and macroscopic particle distribution data can help predict in vivo transport efficiency and targeted accumulation [113]. The application of multi-scale models not only improves the prediction accuracy of the entire delivery system at all levels, but also provides theoretical guidance and an experimental optimization basis for the design of bioactive substance delivery systems in foods with liver enrichment and functionality.

The commonly used machine learning methods in this field include random forest (RF), support vector machine (SVM), gradient enhancement algorithms (such as XGBoost) and artificial neural network (ANN), which are very suitable for capturing nonlinear relationships in complex conveying systems. In these models, the input features usually include material properties (such as particle size, ζ potential, composition), formulation parameters, and processing conditions, while the output variables include encapsulation efficiency, release kinetics, bioavailability, and cell absorption performance. Model performance is usually evaluated using statistical indicators such as R^2^, RMSE, and cross-validation techniques. In addition, external validation using independent data sets is essential to ensure the predictive reliability of the model. Despite these advantages, the current machine learning applications in food-grade conveying systems are still limited by limited data availability, lack of standardized data sets, and variability of experimental conditions, which may affect the repeatability and versatility of the model.

### 6.2. Intelligent Optimization Cycle of Food-Grade Carrier Based on Machine Learning

In the research of food-grade delivery systems, the closed-loop optimization strategy combining experimental data and computational prediction based on ML has become an important development direction of process optimization in the food industry. High-throughput experimental and industrial production data can be fed back to the model in real time to update and optimize calculation parameters, thereby improving prediction accuracy and identifying key factors affecting carrier performance. For example, Ortiz-Perez et al. achieved a significant improvement in carrier performance through ML guidance only in two ML-guided iterations [114].

Combined with multi-scale modeling, ML can play a key role in food formulation design. By simulating the gastrointestinal digestion behavior, shelf life and active substance release kinetics of the carrier in different food matrices, the precise regulation of the delivery system function can be achieved while reducing the experimental cost. For example, Bao et al. introduced a data-driven workflow that combines active learning with experimental automation, and generates data with high-throughput experiments and optimizes them with ML models, which can be used for rapid screening and optimization of nanopreparation performance [115]. Ros et al. used ML algorithm for virtual verification and parameter optimization, which can significantly shorten the development cycle of the new delivery system. It can not only improve the efficiency of carrier design, but also realize the precise regulation of the function of the delivery system under the premise of ensuring food safety [116]. Importantly, this intelligent optimization cycle significantly reduces the number of trial and error experiments, shortens the product development timeline, and lowers costs—key factors for the successful commercialization of functional foods in the competitive food industry.

In summary, the design of ML-driven food-grade oral delivery system integrates the physical and chemical properties, structural parameters and digestive behavior data of materials. These data can be used to construct a multi-scale structure–function prediction model to realize the dynamic simulation of the carrier in the processes of gastrointestinal digestion, transmembrane absorption and liver enrichment. Combined with the intelligent optimization cycle of high-throughput experiments and industrial production data, the particle size, encapsulation efficiency and processing stability of the carrier can be accurately controlled, and the efficiency of functional food formulation development and industrial production consistency can be improved. This strategy not only optimizes the delivery performance and nutritional intervention effect, but also provides technical support for the safe and effective application of liver-protecting active substances in food, and promotes the development of the delivery system in the direction of intelligence and industrialization.

## 7. Current Challenges and Future Development Directions

**Figure 8:**
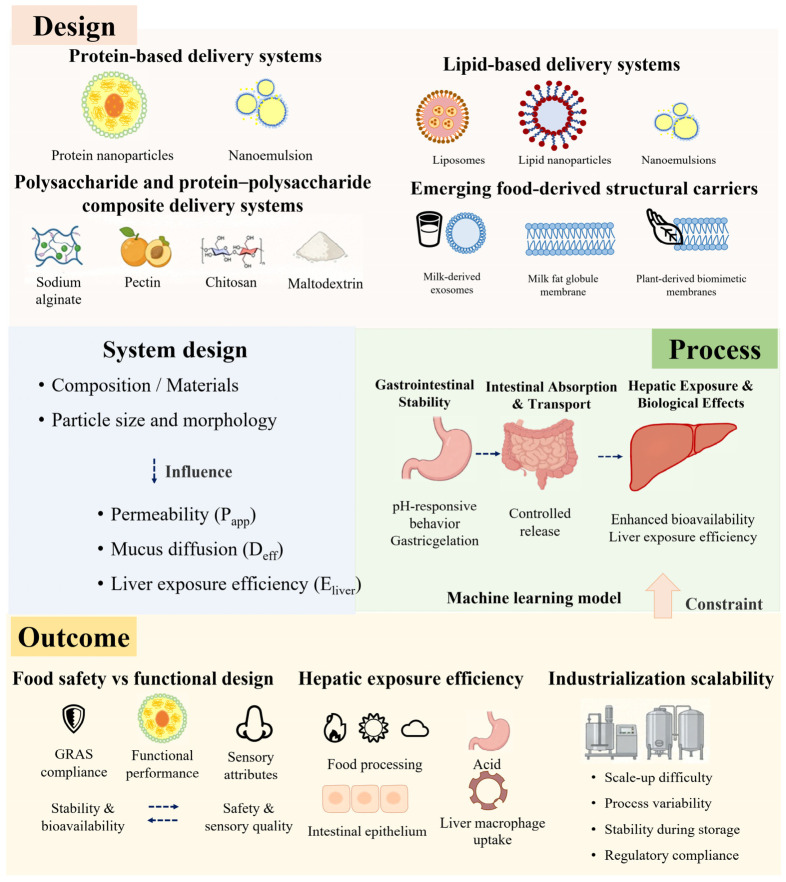
The multi-scale integrated framework of food-grade carriers from structural design to liver exposure: coupling gastrointestinal behavior, transport process, biological effects and machine learning optimization, and subject to safety and industrialization constraints.

Although the current food-grade carrier has established a preliminary research framework around “component and particle size design–gastrointestinal stability–absorption transport–liver exposure–biological effects”, it still remains at the level of segmented optimization and qualitative correlation, and lacks a cross-scale quantitative integration and data-driven closed-loop system that takes into account the balance between food safety and functional design, the mechanism of liver exposure efficiency improvement, and the feasibility of industrialization, which leads to the current key challenges and future development directions in terms of safety–functional trade-offs, liver exposure bottlenecks, and large-scale applications (Figure 8).

### 7.1. Balance Between Food Safety and Functional Design of Delivery Systems

A central challenge for food-grade delivery systems is the quantitative balance between functional performance (e.g., bioavailability, release kinetics) and food restrictions (e.g., GRAS compliance, sensory impact and shelf-life stability).

There is an essential difference between food-grade and drug-grade delivery systems, and this difference has a profound impact on the design strategy of food-source carriers for liver enrichment applications. Food-grade carriers must strictly adhere to GRAS standards and emphasize long-term intake safety and biocompatibility. Therefore, the core challenge lies in balancing functional performance (e.g., stability, bioavailability) with food safety requirements (e.g., raw material compliance, processing compatibility) and sensory attributes (e.g., taste, flavor, shelf life). Optimizing carrier structure and formulation is essential to reconcile stable delivery with food quality enhancement. Recent studies have shown that by constructing a nano-delivery system based on food-grade materials, the stability and bioavailability of bioactive substances such as polyphenols and carotenoids in the gastrointestinal environment can be significantly improved, and sustained health promotion can be achieved [32,117]. The carrier can maintain stability in the gastrointestinal environment and achieve controlled release and absorption of bioactive substances, thereby improving bioavailability while meeting food safety and long-term intake needs [118]. At the same time, the delivery system may also have a combined support effect on liver metabolism and antioxidant defense through a multi-pathway regulatory mechanism [119].

In addition, in the actual development, the food delivery system not only needs to consider the stability and delivery efficiency of bioactive substances, but also must take into account the food processing and consumption attributes. How to achieve a balance between food taste, flavor, shelf-life stability and functional activity has become a core challenge in the design of functional food delivery systems. Thus, optimizing carrier structure and formulation can reconcile stable delivery with food quality enhancement. In summary, the advantages of food carriers in liver enrichment and delivery are reflected in safety and long-term effects. In addition, through low-dose multi-target synergy, they can also achieve more balanced and sustainable health interventions.

### 7.2. The Bottleneck of Hepatic Exposure Efficiency

In the study of food-derived delivery vectors for liver nutrition intervention, improving the hepatic bioavailability of bioactive substances in food-grade delivery systems still faces multiple limiting factors. First of all, in the process of food processing and storage, the delivery system needs to maintain good structural stability to avoid the degradation of active ingredients due to oxidation, and light or processing conditions. In the gastrointestinal digestion environment, the carrier needs to protect the active substances from gastric acid, digestive enzymes and oxidation reactions. Reasonable design of the release kinetics of the carrier, such as the use of pH-sensitive, multi-enzyme response or intestinal-specific degradation strategies to achieve phased release, is a key means to improve liver enrichment and function [120,121].

Secondly, the mucus layer and the intestinal epithelial barrier are the barriers that the oral delivery system must overcome before reaching the liver. Food-grade nano- or microcarriers are often limited by factors such as particle size, surface charge and hydrophobicity when penetrating the mucus layer. Positively charged particles are easily trapped by electrostatic interaction with negatively charged mucus, while overly hydrophobic surfaces may be trapped or aggregated [122,123].

In addition, resident macrophages in the liver have a strong nonspecific phagocytosis of granular carriers. Although this mechanism can prolong the residence time of active substances in the hepatic sinus, it may also reduce their uptake by hepatocytes, thus affecting the exertion of food-derived bioactive substances. Studies have shown that the carrier can be regulated by strategies such as particle size optimization, surface hydrophilic treatment, and receptor recognition ligand modification to improve hepatocyte uptake efficiency and achieve more effective hepatic delivery [124].

In general, the food-grade delivery system is still limited by food processing in improving liver nutrition utilization. The key limiting parameters include particle size distribution, surface charge, the mucus diffusion coefficient (*D*_eff_) and epithelial permeability (*P*_app_), which together determine the exposure efficiency of the liver (*E*_liver_). Through the comprehensive optimization of carrier particle size, surface properties and structural stability, it is expected to achieve a synergistic balance between food processing suitability, digestive stability and bioavailability, thus providing an important theoretical basis for precise nutritional intervention in functional foods.

### 7.3. Industrial Viability of Food-Grade Delivery Systems

In the process of transforming the food-derived delivery system into practical application, industrial scale production, food processing adaptability and regulatory compliance are the key factors affecting its industrialization.

Firstly, in the large-scale production of the food industry, the delivery system needs to ensure the consistency of particle size distribution, structural stability and encapsulation efficiency of bioactive substances [125]. At present, the emulsion method, self-assembly and spray drying technology commonly used in the laboratory have gradually transitioned to the common processing technology in the food industry. For example, high-pressure homogenization or microfluidization technology can be used for large-scale preparation of nanoemulsions to improve the dispersion stability of fat-soluble active substances in beverage systems [126]. Spray drying technology is widely used in the preparation of polyphenol or carotenoid microcapsule powder, which is convenient for stable addition and storage in functional foods [127]. Extrusion molding technology can integrate protein or polysaccharide-based delivery systems into cereal-based or meal replacement products to achieve functional delivery in solid foods [128]. Microfluidic technology is an emerging preparation method. With its excellent fluid control ability and structural designability, it provides a new paradigm for the precise and continuous preparation of food-grade carriers. It can realize the construction of the carrier with highly uniform particle size and a complex structure [129]. However, the current application of this technology in the food field still faces problems such as low processing flux, high equipment investment costs, and lack of systematic research on the suitability of complex food matrices. There is still a certain distance from laboratory prototype to industrial scale production. This requires a balance between the stability of the delivery architecture and the process scalability by optimizing process parameters (such as shear force, temperature and moisture conditions).

Despite these advances, some technical challenges still limit the industrial scalability of food-grade conveying systems. It is difficult to maintain structural integrity and functional performance during large-scale processing, in which shear force, heat treatment and moisture changes will significantly change the performance of the carrier. In addition, achieving consistent particle size distribution and encapsulation efficiency in different batches remains challenging, especially for complex composite systems. Another limitation is the compatibility of the delivery system with different food matrices, as interactions with proteins, lipids and other food components may affect stability and release behavior. In addition, although emerging technologies such as microfluidics are expected to achieve precise structural control, they are currently limited by low production volume and high operating costs, hindering their industrial applications.

Secondly, the design of the delivery system needs to be matched with the specific food product form to ensure good stability and functional performance during food processing and consumption. For example, nanoemulsions with good transparency and dispersion stability are suitable for transparent functional beverages or plant beverage systems [130]. Microcapsule powder can be widely used in meal replacement powder or solid beverages to improve the stability of active ingredients during storage and processing [131]. By combining the delivery system with different food forms, the stable delivery and sustained release of bioactive substances can be achieved while ensuring the taste and appearance quality of the products.

In addition, food regulatory requirements and consumer acceptance are also important factors in the industrialization of functional food. Protein, polysaccharide and lipid materials used to construct delivery systems need to meet the requirements of food safety regulations. The construction of food-grade delivery systems must be based on compliant food raw materials to ensure a reliable source and process traceability. At the same time, in food labeling and product promotion, it is necessary to clearly mark the source of carrier raw materials and functional information in order to improve product transparency and consumer trust, thereby enhancing consumer acceptance of liver-protecting foods.

From a regulatory perspective, industrial applications of food-grade conveying systems face additional challenges. Unlike drug delivery systems, food-grade carriers must comply with strict food safety regulations, such as GRAS and region-specific food additive regulations. The use of novel nanostructured materials in food may increase regulatory uncertainty, particularly with regard to their long-term safety and labeling requirements. In addition, consumers’ perceptions of nano or structurally modified food systems may affect market acceptance. Therefore, the future development of food-grade delivery systems should not only consider functional performance, but also consider regulatory compliance, risk assessment and transparent communication to improve consumer trust and promote commercialization.

Finally, the stability of food-grade delivery systems is also an important factor affecting the commercialization of products. In the actual food system, temperature, light and oxygen conditions may affect the stability of the delivery structure and the preservation of active substances [132,133]. Therefore, in the process of product development, it is necessary to systematically evaluate the stability of the delivery system under different storage conditions (such as room temperature or cold storage, dark or aerobic environment) to ensure that functional foods maintain stable active substance content and functional effects throughout their shelf life.

In summary, the industrial application of food-derived delivery systems not only depends on the improvement of the stability and bioavailability of active ingredients: it also needs to be coordinated with the food industry production process, product form design, regulatory requirements and shelf-life stability. By systematically optimizing material selection, processing technology and product development strategies, the delivery system can be transformed from laboratory research to functional food products, providing a more feasible application path for liver health-related nutritional interventions. Future research should focus on linking process parameters (such as shear, temperature, and humidity) to structural stability and functional performance through data-driven optimization and amplification modeling.

## 8. Conclusions

In summary, food-grade oral delivery systems represent a promising strategy that can improve the stability, bioavailability, and liver exposure of food-derived bioactive substances, thereby enabling more effective nutritional interventions for liver oxidative stress. This review systematically integrates current advances by linking carrier design strategies to gastrointestinal behavior, intestinal transport, and hepatocyte uptake, and further links these mechanisms to practical considerations such as food processing compatibility and industrial scalability. This work systematically correlates carrier structure, digestion kinetics, and liver-targeting results. Compared with traditional studies that focus on encapsulation performance or in vitro biological activity, this review provides a more application-oriented perspective. In addition, the combination of emerging methods such as machine learning-aided design and food-grade material innovation provides a new direction for the rational optimization of conveying systems.

However, there are still some limitations in the current research field. First, most existing studies are based on in vitro digestion models and cell experiments, with limited validation in in vivo or in human studies, which limits the reliability of conclusions under realistic dietary conditions. Secondly, due to the lack of standardized assessment framework and unified liver exposure indicators, it is difficult to compare the results of different studies. Third, many delivery systems are still being developed on a laboratory scale, without fully considering process adaptability, sensory characteristics, and long-term stability of real food systems. In addition, the doses of bioactive substances used in experimental studies are usually higher than daily dietary intake, which may limit their practical application.

Key priorities include: (1) developing food-grade, biocompatible delivery carriers with improved performance and proven safety for long-term dietary consumption; (2) developing low-cost carriers based on food processing by-products to achieve high-value utilization; (3) optimizing the taste and flavor of the delivery system in functional foods; (4) establishing a standardized evaluation method for food-grade delivery system, and promoting the industrialization development of liver-protecting food. In the future, with the in-depth study of the structure–function relationship and the optimization of production processes, food-grade oral delivery systems will play an important role in promoting the development of the food industry in the direction of precision nutrition and green sustainability.

## Figures and Tables

**Table 1:** Summary of embedding food-grade active substances in delivery carrier systems from different food sources.

Main Raw Materials	Delivery Carriers	Bioactive Substances	Preparation Technology	Advantages	Limitations	References
Whey protein	Nanocomposite	Curcumin	pH-driven self-assembly	Improve the photothermal stability and storage stability; improve the bioavailability	Lack of quantitative correlation between particle structure, gastrointestinal fate, and hepatic exposure	[44]
Whey protein	Emulsions	Curcumin	High-pressure homogenization emulsification	Improve the stability of curcumin emulsion and in vitro digestion bioaccessibility	Limited long-term physicochemical stability and susceptibility to phase separation	[45]
Whey protein	Emulsions	Curcumin	High-pressure homogenization emulsification	Physical and chemical stability, strong antioxidant activity	Lack of in vivo oral bioavailability and actual stability verification	[46]
Whey protein and zein	Nanoparticles	Curcumin	pH-driven self-assembly	Improve the thermal stability, physical stability and redispersibility of curcumin	Lack of absorption efficiency and biological activity after oral administration in vivo	[47]
Whey protein and zein	Nanoparticles	Curcumin	pH-driven self-assembly	Improve the antioxidant activity of curcumin and achieve gastrointestinal controlled release	Indirect hepatic effects difficult to quantify	[48]
Soy protein and zein	Nanoparticles	Curcumin and diosmetin	pH-driven self-assembly	Improve the encapsulation efficiency, loading efficiency and storage stability	Complex formulation	[49]
Soy and whey protein	Microcapsules	Curcumin	Spray drying	Enhanced digestibility and absorption	Lack of in vivo validation and insufficient data on hepatic bioavailability	[50]
Zein and sodium caseinate	Nanoparticles	Curcumin and quercetin	Not studied	Enhance stability and antioxidant activity; improve bioaccessibility in the gastrointestinal tract	Complex interactions difficult to control	[51]
Coconut protein	Nanoparticles	Curcumin	Self-assembly	Antioxidant activity and sustained release capacity	Lack of performance comparison with other plant protein nanocarriers	[52]
Lactobionic acid	Nanoparticles	Astaxanthin	Self-assembly-solvent evaporation method	Cells uptake and scavenge free radicals, protect mitochondria	High PDI	[6]
Lactobionic acid	Nanocomposite	Astaxanthin	Self-assembly	Good water solubility and pH-responsive ability	Complex preparation process	[53]
Lactobionic acid, and sodium alginate	Nanocomposite	Astaxanthin	Self-assembly	Improve the stability and solubility of astaxanthin	Oxidation sensitivity	[54]
Finger citron polysaccharide	Nanoparticles	Luteolin	Self-assembly	Improve solubility and bioavailability	Encapsulation rate medium	[55]
Sodium alginate	Microgels	*Ginkgo biloba* leaf polysaccharide	Inverse emulsion method	Regulating intestinal microbiota; activate the antioxidant pathway	Large particle size, lack of actual food validation	[56]
Bovine bone gelatin	Pickering emulsion	Curcumin	pH-driven self-assembly	High encapsulation efficiency, antioxidant synergistic effect	Poor emulsion stability, strong pH dependence	[57]
Angelica sinensis polysaccharide	Nanoparticles	Curcumin	Self-assembly	Higher solubility, good light stability and sustained release of curcumin within 72 h	Lack of systematic evaluation linking release behavior to in vivo bioavailability and hepatic delivery	[58]
Galactooligosaccharides and whey protein	Nanoparticles	Astaxanthin	Not studied	Double targeting synergistic effect, increase of enzyme activity in vivo	More complex preparation	[59]
Lentinus edodes mycelia polysaccharide and bovine lactoferrin	Nanocomposite	Not studied	Not studied	Reduce oxidative stress, inhibit apoptosis and promote glucose uptake	Lack of compositional clarity and absence of systematic characterization and reproducibility evaluation	[60]
Haematococcus pluvialis protein and galactose	Nanoparticles	Curcumin	Anti-solvent precipitation	Improve the stability of curcumin under strong acid, salt ion and ultraviolet irradiation conditions	Larger particle size	[61]
Soy protein and pectin	Nanocomplexes	Curcumin	pH-driven self-assembly	Composite encapsulation, sustained release stability	Partial aggregation, lack of in vivo verification	[62]
Ovalbumin–fucoidan	Nanoparticles	Nicotinamide mononucleotide	pH-driven self-assembly	Improve the anti-oxidative stress and anti-aging ability of nicotinamide mononucleotide	Limited understanding of in vivo delivery efficiency and targeting behavior	[63]
Soybean phospholipids	Liposomes	Lycopene and nicotinamide mononucleotide	Thin-film ultrasound method	High encapsulation efficiency, synergistic multi-mechanism protection	Complex preparation process	[64]
Egg yolk lecithin	Liposomes	Collagen	Thin-film dispersion method	Improve the stability of collagen to high temperature, pH and ionic strength; enhance the stability and biological function of collagen	Limited understanding of in vivo delivery efficiency	[65]
Egg yolk phospholipids	Liposomes	Probiotic	Thin-film dispersion method	Improve the stability and bioavailability of probiotics	Limited protection against harsh gastrointestinal environments	[66]
Soy phosphatidylcholine and cholesterol	Liposomes	Curcumin	Not studied	Excellent lysosomal targeting efficacy	Complex preparation process	[67]
Soy phosphatidylcholine and cholesterol	Liposomes	Quercetin	Film dispersion-homogenizing method	Improve the solubility and bioavailability of quercetin	Complex model composite factors	[68]
Soy lecithin and Cholesterol	Liposomes	Baicalin	Film rehydration method	Reduce liver inflammatory cell infiltration and production of pro-inflammatory mediators	Lack of long-term stability	[69]
Egg yolk lecithin and cholesterol	Liposomes	Chrysin	Thin-film dispersion method	Improve bioavailability	Lack of targeted verification	[70]
Carboxymethyl chitosan	Liposomes	Fish oil	Not studied	Improve the oxidation stability and application applicability of fish oil	Not studied	[71]
Lecithin; Tween-80	Microemulsion	Docosahexaenoic acid and curcumin	Ultrasonic emulsification	Improve the bioavailability of flavin and docosahexaenoic acid; reduce liver fat deposition	Not studied	[72]
Pepper	Exosome	Curcumin	Differential centrifugation	Improve solubility and utilization	Lack of in vivo verification	[73]
Milk	Exosome	Epicatechin gallate	Differential centrifugation	Enhanced neuroprotective effect; anti-apoptosis and anti-phagocytosis	Limited understanding of in vivo delivery efficiency and targeting behavior	[74]

## Data Availability

The original contributions presented in this study are included in the article. Further inquiries can be directed to the corresponding authors.

## Supplementary Materials

The following supporting information can be downloaded at: https://www.mdpi.com/article/10.3390/foods15101713/s1, Table S1: Summary of studies on food-grade delivery systems for bioactive substances. PRISMA 2020 Main Checklist is presented as a Supplementary Material.

## Abbreviations

The following abbreviations are used in this manuscript:

ROS	reactive oxygen species
GRAS	Generally Recognized as Safe
*P*_app_	apparent permeability coefficient
*D*_eff_	effective diffusion coefficient
*E*_liver_	liver exposure efficiency
SCFAs	short-chain fatty acids
LNPs	lipid nanoparticles
HPLC	high-performance liquid chromatography
LC–MS/MS	liquid chromatography–tandem mass spectrometry
ML	machine learning
RF	random forest
SVM	support vector machine
ANN	artificial neural network
