# Supplementary material for: Food-Grade Delivery Systems for Hepatoprotective Functional Foods: From Rational Design and Delivery Mechanisms to Industrial Processing and Nutritional Intervention
Source: Foods. 2026 May 13;15(10):1713. doi: 10.3390/foods15101713 (PMC13206631; doi:10.3390/foods15101713)
Supplement: Supplementary file 1 [file foods-15-01713-s001.zip › Table S1.pdf]

Table S1. Summary of studies on food-grade delivery systems for bioactive substances.

No.	Study type	Main raw materials	Delivery carriers	Bioactive substances	Preparation technology	Key Findings
1	Review					Regulate the gut-liver axis using plant foods and their active ingredients
2	Review					Use delivery strategies such as nanofibers to improve their low bioavailability and promote the development of functional foods
3	Article	Chlorella vulgaris 211/8k	Nanoparticles	Carotenoid	Self-assembly method	Reduce mitochondrial ROS release and sensitivity to oxidative stress
4	Review					Apply the food-grade nano-delivery system as a feasible strategy for the high-value application of astaxanthin in functional foods
5	Article	Glycogen, α-lipoic acid, lactobionic acid	Nanoparticles	Resveratrol	Self-assembly method	Achieve liver targeting and redox-responsive release behavior
6	Article	Lactobionic acid	Nanoparticles	Astaxanthin	Self-assembly-solvent evaporation method	Improve the cell uptake rate, antioxidant capacity and liver-specific accumulation of astaxanthin

7	Review					Strategies to improve the bioavailability of antioxidant peptides, including nanocarriers
8	Review					Use polyphenols to play a therapeutic role in liver injury by regulating the level of reactive oxygen species
9	Article	Proanthocyanidins, sea cucumber peptide	Nanoparticles	Astaxanthin	Self-assembly method	Ensure high drug loading, pH responsiveness, and excellent temperature/UV stability
10	Review					Tailor the preparation and preparation technology of the delivery system according to the specific characteristics of bioactive peptides
11	Review					Use the oral delivery system to improve the solubility, stability, and permeability of the peptide and overcome its weakness of poor chemical/physical stabilit
12	Review					Investigate the influence mechanism of food matrix on the behavior of delivery carriers
13	Review					Overcome the limitations of traditional delivery tools and

		achieve targeted delivery using nanotechnology
14	Review	Overcome oral delivery barriers of functional proteins
15	Review	Use protein-polysaccharide complex as an ideal material for constructing a nutrition and health care product delivery system, taking advantage of its nutrition, safety, and physicochemical properties
16	Review	Summarize the structure, preparation methods, and incorporation strategies of liposomes in various matrices, focusing on food-grade components
17	Review	Release payloads to systemic circulation using lipid nanocarriers through paracellular pathways, lymphatic absorption, endocytosis/transcytosis, or membrane fusion

18	Review			Adjust the solubility, stability, and intestinal absorption using food colloid delivery carriers; enhance the nutritional added value of functional food, improve sensory characteristics, and prolong shelf life
19	Review			Study the biogenesis, function, extraction, and characterization methods of exosomes, available resources, and the design concept of biomimetic nanocarriers
21	Review			Design accurate oral drug delivery system targeting strategies and delivery principles to overcome barriers and improve therapeutic effects
22	Review	Proteins and peptides		Evaluate the advantages and disadvantages of various strategies and emphasize the collaborative integration of multiple methods to effectively overcome multiple barriers
23	Review		Polyphenol	Integrate microbiome and polyphenol research to summarize and contextualize the

						health benefits of dietary polyphenols
24	Review					Emphasize the key role of metabolic types in health regulation via polyphenol-microbiota coevolution
25	Review					Apply liver organoids in functional food evaluation and food safety risk assessment
26	Review					Classify mucosal adhesion polymers and mucosal adhesion delivery systems designed for nutritional and health products using the system
27	Review					Use nanotechnology (nano-food) to improve the nutritional and sensory characteristics of food and improve the bioavailability of bioactive compounds
28	Review					Apply nanotechnology in the food industry to improve texture, flavor, bioavailability, and shelf life
29	Article	Prebiotic fibers polydextrose and inulin	Microcapsule	Sugar beet anthocyanins and	Freeze-drying method	Achieve maximum release and stability in the gastric phase

				phenolic compounds		
30	Review					Establish a robust quantitative structure-permeability relationship model with enhanced predictive ability
31	Review					Use food-grade pH-sensitive nanoparticles to improve the stability of components in the stomach and control their release in the intestine, thereby enhancing absorption and bioavailability
32	Article	Tween 80, lecithin	Nanodiscs	Not studied	Self-assembly method	Ensure superior packaging performance
33	Article	Polyethylene glycol, concanavalin A, pectin	Nanoparticles	Not studied	Self-assembly method	Develop nanoparticles to improve the bioavailability of oral biological agents
34	Article	Lipid	Liposomes	Coenzymum Q10	Microfluidic method	Achieve mitochondrial targeted delivery
35	Review		Emulsion			Prepare the system by simple processing using food-grade ingredients
36	Article	Ovalbumin, amino, polyethylene glycol,	Nanoparticles	Not studied	Self-assembly method	Regulate the mucosal delivery behavior of protein nanoparticles via surface modification

		chitosan, oligonucleotides				
37	Article	Transferrin	Nanoparticles	Not studied	Self-assembly method	Promote the adsorption of endoplasmic reticulum-Golgi related proteins, enhance intracellular transport and exocytosis
38	Article	Diacylglycerol, palmitic acid acetyl, dopamine, polyethylene glycol	Lipid nanoparticle	Linseed cyclopeptide	Not studied	Achieve high permeability to improve anti-inflammatory efficiency
39	Article	Pectin	Nanoparticles	Not studied	Self-assembly method	Improve lipid metabolism
40	Article	Rice peptide	Nanoparticles	Not studied	Self-assembly method	Alleviate obesity, oxidative stress and inflammation in mice with alcoholic liver disease
41	Review	Inulin-type and neoinulin-type fructan				Provide immune regulation, liver protection, and prebiotic activity
42	Review	Polysaccharide		Probiotics		Use optimized particle embedding technology
43	Review					Use short chain fatty acids to affect a variety of physiological processes
44	Article	Lactobionic acid, and sodium alginate	Nanocomposite	Astaxanthin	Self-assembly method	Improve the stability and solubility of astaxanthin
45	Article	Whey protein, gum arabic	Emulsion	Not studied	High pressure homogenization method	Enhance oxidation stability, elastic dominant properties,

						apparent viscosity, and excellent storage stability
46	Article	Camel casein, bovine whey protein	Nanocomposite	Curcumin	Self-assembly method	Significantly improve the stability under gastrointestinal conditions
47	Article	Sodium alginate	Microgels	Ginkgo biloba leaf polysaccharide	Inverse emulsion method	Regulating intestinal microbiota; Activate the antioxidant pathway
48	Article	Bovine bone gelatin	Pickering emulsion	Curcumin	Self-assembly method	Improve inoxidizability
49	Article	Angelica sinensis polysaccharide	Nanoparticles	Curcumin	Self-assembly method	Achieve higher solubility, good light stability and sustained release of curcumin within 72 hours
50	Article	Galactooligosaccharides and whey protein	Nanoparticles	Astaxanthin	Not studied	Relieve oxidative stress
51	Article	Lentinus edodes mycelia polysaccharide and bovine lactoferrin	Nanocomposite	Not studied	Not studied	Reduce oxidative stress, inhibit apoptosis and promote glucose uptake
52	Article	Haematococcus pluvialis protein and galactose	Nanoparticles	Curcumin	Anti-solvent precipitation	Improve the stability of curcumin under strong acid, salt ion and ultraviolet irradiation conditions
53	Article	Soy protein and pectin	Nanocomplexes	Curcumin	Self-assembly method	Improve the photothermal stability, sustained release and

						maintain antioxidant activity of curcumin
54	Article	Ovalbumin-fucoidan	Nanoparticles	Nicotinamide mononucleotide	Self-assembly method	Improve the anti-oxidative stress and anti-aging ability of nicotinamide mononucleotide
55	Article	Soybean phospholipids	Liposomes	Lycopene and nicotinamide mononucleotide	Thin-film ultrasound method	Inhibit oxidative stress and inflammation, and regulate intestinal microflora
56	Article	Egg yolk lecithin	Liposomes	Collagen	Thin film dispersion method	Improve the stability of collagen to high temperature, pH and ionic strength; Enhance the stability and biological function of collagen
57	Article	Egg yolk phospholipids	Liposomes	Probiotic	Thin film dispersion method	Improve the stability and bioavailability of probiotics
58	Article	Soy phosphatidylcholine and cholesterol	Liposomes	Curcumin	Not studied	Achieve excellent lysosomal targeting efficacy
59	Article	Soy phosphatidylcholine and cholesterol	Liposomes	Quercetin	Film dispersion-homogenizing method	Improve the solubility and bioavailability of quercetin
60	Article	Soy lecithin and Cholesterol	Liposomes	Baicalin	Film rehydration method	Reduce liver inflammatory cell infiltration and production of pro-inflammatory mediators
61	Article	Egg yolk lecithin and cholesterol	Liposomes	Chrysin	Thin film dispersion method	Reduce the accumulation of lipid in the liver, reduce liver injury,

						reduce the production of free radical oxygen
62	Article	Carboxymethyl chitosan	Liposomes	Fish oil	Not studied	Improve the oxidation stability and application applicability of fish oil
63	Article	Lecithin; Tween-80	Microemulsion	DHA and curcumin	Ultrasonic emulsification	Improve the bioavailability of flavin and DHA; Reduce liver fat deposition
64	Article	Milk	Exosome	Epicatechin gallate	Differential centrifugation	Enhanced neuroprotective effect; Anti-apoptosis and anti-phagocytosis
65	Article	Phosphatide	Porous microgels	Lycopene and nicotinamide mononucleotides	Not studied	Provide good stability and release characteristics
66	Article	Lecithin	Liposomes	Collagen	Thin film dispersion method	Enhance stability and biological function
67	Article	Phospholipids, inulin	Liposomes	Lactobacillus rhamnosus	Not studied	Improve the stability and bioavailability of probiotics
68	Article	Phospholipids, inulin	Liposomes	Curcumin	Film rehydration method	Achieve better lysosomal targeting and better biocompatibility
69	Article	Phospholipids, inulin	Liposomes	Quercetin	Film dispersion-homogenizing method	Achieve higher biological activity and bioavailability, and significantly reduce the liver

						index and pathological changes of damaged liver tissue
70	Article	Phospholipids, inulin	Liposomes	Baicalin	Thin film dispersion method	Improve bioavailability
71	Article	Phospholipids	Liposomes	Chrysin	Thin film dispersion method	Reduce lipid accumulation, reduce liver injury, reduce free radical oxygen production
72	Article	Phospholipids	Liposomes	Fish oil	Thin film dispersion method	Reduce NF-κB activation, play a role in liver protection.
73	Article	Oil, surfactant	Microemulsion	Curcumin, docosahexaenoic acid	Ultrasonic emulsification method	Improve bioavailability
74	Article	Zein and Sodium Caseinate	Nanoparticles	Curcumin and quercetin	Not studied	Enhance stability and antioxidant activity; Improve bioaccessibility in the gastrointestinal tract
75	Article	Milk	Exosomes	Epicatechin gallate	Ultrasonic method	Improve cell viability, reduce reactive oxygen species, reduce apoptosis rate
76	Article	Buckwheat	Nanoparticles	Polyphenols	Not studied	Prevent oxidative stress
77	Article	Soybean glycine, β-galactose	Nanoparticles	Not studied	Self-assembly method	Form smaller and more stable nanoparticles
78	Article	Soy protein	Nanoparticles	Curcumin	Self-assembly method	Ensure high compatibility and cellular antioxidant accessibility
79	Article	Casein	Microcapsule	Anthraquinones	Ultrasonic assisted spray drying method	Improve bioavailability

80	Article	Insoluble soybean peptide aggregates	Nanoparticles	Curcumin	Self-assembly method	Excellent storage stability and antioxidant activity
81	Article	Whey protein isolate	Nanocomposite	Astaxanthin	Emulsification-evaporation method	Increase the apparent permeability coefficient of Caco-2 cells
82	Article	Whey protein isolate	Emulsion	Astaxanthin	Emulsification-evaporation method	Improve the bioavailability of astaxanthin
83	Article	α-lactalbumin	Nanocomposite	Curcumin	Self-assembly method	Improve the stability of curcumin to ultraviolet light
84	Article	Cod protein	Microcapsule	Vitamin D	Not studied	Improve bioaccessibility
85	Article	Soybean lecithin, chitosan, sodium alginate	Microcapsule	Astaxanthin	Freeze-drying method	Achieve slow release characteristics and improve the chemical stability of astaxanthin
86	Article	Pepper	Exosome	Curcumin	Differential centrifugation	Improve solubility and utilization
87	Article	Chitosan, pectin	Nanoparticles	Astragalus polysaccharid	Not studied	Ensure good physical and chemical stability, pH-responsive release, and slow liver steatosis
88	Article	Whey protein	Nanocomposite	Curcumin	Self-assembly method	Improve the photothermal stability and storage stability; Improve the bioavailability
89	Article	Whey protein	Emulsion	Curcumin	High-pressure homogenization emulsification	Improve the stability of curcumin emulsion and *in vitro* digestion bioaccessibility
90	Article	Whey protein	Emulsion	Curcumin	High-pressure homogenization emulsification	Improve the antioxidant activity of curcumin

91	Article	Whey protein and zein	Nanoparticles	Curcumin	Self-assembly method	Improve the thermal stability, physical stability and redispersibility of curcumin
92	Article	Whey protein and zein	Nanoparticles	Curcumin	Self-assembly method	Improve the antioxidant activity of curcumin and achieve gastrointestinal controlled release
93	Article	Soy protein and zein	Nanoparticles	Curcumin and diosmetin	Self-assembly method	Improve the encapsulation efficiency, loading efficiency and storage stability
94	Article	Soy and whey protein	Microcapsule	Curcumin	Spray drying	Achieve higher proteolysis rate, curcumin release rate, digestion stability and in vitro intestinal absorption rate
96	Article	Coconut Protein	Nanoparticles	Curcumin	Self-assembly method	Improve the solubility and antioxidant activity in aqueous solution; Achieve good sustained-release performance.
97	Article	Lactobionic acid	Nanocomposite	Astaxanthin	Self-assembly method	Provide good water solubility and pH-responsive ability
98	Article	Finger citron polysaccharide	Nanoparticles	Luteolin	Self-assembly method	Improve the water solubility and bioavailability of lutein; Reduce lipid accumulation and oxidative stress
99	Article	Mushroom	Exosomes	Curcumin	Not studied	Improve the stability of curcumin
100	Article	Cordyceps extracellular polysaccharide, selenite	Nanoparticles	Not studied	Not studied	Protect mitochondrial membrane potential

101	Review			Polyphenols		Use the nano-delivery system to improve the solubility and stability of polyphenols and play a sustained release role
102	Review					Regulate gut microbiota and its metabolites as a potential strategy for liver health
103	Article	Oleic acid, stearic acid	Lipid nanoparticle	Not studied	Ultrasound methods	Improve oral bioavailability
104	Article	Pectin, sodium caseinate	Nanoparticles	Resveratrol, curcumin	Solvent evaporation combined with electrostatic deposition method	Enhance antioxidant activity
105	Review			Polyphenol		Use new strategies such as nanoencapsulation and synergistic combination to improve the bioavailability of polyphenols
106	Article	Alginate, inulin, chitosan	microballoon	Quercetin	Ion gelation method	Promote the production of short-chain fatty acids
107	Article	Buckwheat	Nanoparticles	Polyphenol	Not studied	Regulate energy metabolism, lipid synthesis and lipid oxidation
108	Article	Sodium alginate, chitosan	microballoon	Quercetin	Ion gelation method	Increase antioxidant levels by more than 7 times
109	Review					Use nanomaterials combined with AI to achieve accurate detection, targeted delivery, and

						improve the bioavailability of food components
110	Review					Employ nanocarriers based on biopolymers and lipids to achieve higher stability and retention than traditional systems
111	Article	Various nanomaterials	Nanoparticles	Curcumin	Not studied	Apply machine learning for predictive toxicology and curcumin nanocarrier optimization
112	Review					Use artificial intelligence to enhance permeability, targeting, and controlled release capabilities through deep learning, data analysis, and optimized carrier design to overcome these obstacles
113	Review					Utilize development software, force fields, and technologies in MD simulations to explore the delivery of bioactive substances
114	Article	Not studied	Nanoparticles	Not studied	Microfluidic method	Achieve a high uptake rate

115	Article	12 different excipients	Nanoparticles	Not studied	Active learning robot system	Use the data-driven workflow to significantly improve solubility, obtain small and uniform particles, and remain stable during storage
116	Review					Use computer learning to accelerate the development of delivery carriers, reduce the experimental burden, and open up new design space
117	Article	zein	Nanoparticles	Resveratrol, coenzyme Q10	Emulsification-evaporation method	Improve co-delivery efficiency and chemical stability
118	Review	carrageenan				Use the carrageenan-based delivery system to improve the stability and bioavailability of bioactive components
119	Review		Microcapsule	carotenoid		Apply carotenoid microencapsulation technology to improve storage stability and bioavailability
120	Review	β-cyclodextrin				Form inclusion complexes to protect bioactive substances and regulate release
121	Article	V-type single spiral starch	Nanoparticles	Quercetin		Provide gastrointestinal degradation protection and relieve chronic inflammation

122	Article	Polysaccharides extracted from intestinal worms	Nanoparticles	Polysaccharide	Self-assembly method	Provide antioxidant activity and water absorption/retention capacity
123	Review		Micelles			Use micelles to encapsulate charged biomolecules, improve their stability and solubility, and maintain or enhance biological activity
124	Review		Nanoparticles			Understand the physiology of the hepatic sinusoid and nanoparticle-hepatocyte interaction to optimize the liver-targeted delivery system, taking advantage of the liver's natural tendency for nanoparticles
125	Review		zein			Compare different techniques for preparing zein granules, optimize process parameters, evaluate advantages and disadvantages, and determine the final application
126	Review	Protein				Achieve release regulation of the expandable matrix by appropriately combining structural relaxation, expansion, and erosion mechanisms

127	Article	Chitosan, seaweed polyphenols	Pickering Emulsion	β-carotene	spray drying method	Provide three-dimensional network and antioxidant activity
128	Review					Investigate interactions between starch and other food components during extrusion
129	Review				Microfluidic method	Use microfluidic technology to accurately control the structural design and production of the conveying system and achieve controlled release
130	Article	Fish gelatin, transglutaminase, sunflower seed oil	emulsion	Not studied	Microfluidic method	Ensure emulsion stability and physical limit stability
131	Article	Natural casein	micelle	Curcumin	Self-assembly method	Achieve antioxidant effect and improve bioavailability
132	Article	Mediumaltitude bombing chain triglyceride	Emulsion	Curcumin	High pressure homogenization method	Improve water dispersibility and chemical stability
133	Article	Skim milk powder, Tween 80, maltose dextrin,	Microcapsule	walnut oil	Spray drying method	Achieve the highest encapsulation efficiency and the lowest oil surface coverage
